# Mortality after emergency presentation: evidence from a cohort of 929 378 Medicare beneficiaries with common cancers diagnosed 2008–2017

**DOI:** 10.1093/jnci/djag236

**Published:** 2026-07-14

**Authors:** Caroline A. Thompson, Yuelin He, Sarah E. Soppe, Xueer Zhang, Megan A. Mullins, Matthew E. Barclay, Nicholas Pettit, Allison W. Kurian, Ellis C. Dillon, Sandi Pruitt, Georgios Lyratzopoulos

**Affiliations:** 1 Department of Epidemiology, Gillings School of Global Public Health, University of North Carolina, Chapel Hill, NC, United States; 2 Lineberger Comprehensive Cancer Center, University of North Carolina at Chapel Hill, Chapel Hill, NC, United States; 3 Department of Health Economics, Systems, and Policy, Peter O’Donnell Jr. School of Public Health, UT Southwestern Medical Center, Dallas, TX, United States; 4 Harold C. Simmons Comprehensive Cancer Center, UT Southwestern Medical Center, Dallas, TX, United States; 5 Department of Behavioural Science and Health, Epidemiology of Cancer Healthcare and Outcomes group, Institute of Epidemiology and Health Care, University College London, London, United Kingdom; 6 Department of Emergency Medicine, Indiana University School of Medicine, Indianapolis, IN, United States; 7 Departments of Medicine and of Epidemiology and Population Health, Stanford University School of Medicine, Stanford, CA, United States; 8 Center on Aging, University of Connecticut, Farmington, CT, United States; 9 Department of Public Health Sciences, University of Connecticut, Farmington, CT, United States; 10 Department of Social and Behavioral Sciences, Peter O’Donnell Jr. School of Public Health, UT Southwestern Medical Center, Dallas, TX, United States

## Abstract

**Background:**

Emergency presentation (EP) for cancer diagnosis is associated with higher mortality, but prior research has focused largely on select cancer types or non-US populations. We estimated EP prevalence and associated mortality across 16 cancers in a national Medicare cohort.

**Methods:**

Using SEER-Medicare data, we identified patients with 16 high-burden cancers diagnosed from 2008 to 2017. EP was defined by an emergency department (ED) claim within 30 days before the index cancer claim and subtyped as inpatient EP (emergency hospitalization) or outpatient EP (after ED discharge). We estimated survival probabilities and mortality risk ratios (RRs) adjusted for demographics, tumor characteristics, comorbidity, frailty, and health-care utilization.

**Results:**

Among 929 378 patients, 28% were EPs (22% inpatient, 6% outpatient). EP prevalence ranged from <10% (breast, prostate) to >40% (liver, lung, stomach, colon, ovarian, pancreatic cancers). One-year survival was 81% for non-EPs, 60% for outpatient EPs, and 36% for inpatient EPs. Compared with non-EPs, inpatient EPs had nearly 4-fold higher 30-day adjusted mortality (RR = 3.88, 95% CI = 3.81 to 3.96), ranging from 2.1 (pancreas) to 6.3 (lymphoma). Among 90-day survivors, inpatient EPs had 51% higher 1-year mortality (RR = 1.51, 95% CI = 1.49 to 1.52), ranging from 1.1 (pancreas) to 2.1 (lymphoma). Outpatient EPs showed intermediate risk (1-year mortality RR = 1.28, 95% CI = 1.26 to 1.30).

**Conclusion:**

EP is common and predicts persistently elevated mortality in older US adults after comprehensive covariate adjustment. Associations were strongest for cancer types underrepresented in prior research, including hematological malignancies and breast, prostate, and bladder cancers. Routine EP monitoring could improve risk stratification at diagnosis and guide efforts to reduce avoidable emergency presentations.

## Introduction

Although cancer is rarely definitively diagnosed in the emergency department (ED), an ED visit may be the first encounter where malignancy is suspected. These presentations can lead to immediate hospitalization and treatment or referral for urgent outpatient care.^1^ Affected patients may have severe symptoms, high frailty or comorbidity burden, limited access to routine care, or acute conditions during which a cancer is discovered incidentally.^2^ *Emergency presentation* (EP)—a new cancer diagnosis after ED contact—is globally recognized as a marker of advanced disease and suboptimal diagnostic pathways. EP is consistently associated with excess short-term mortality, even after adjusting for stage and other prognostic factors, suggesting it may signal worse prognosis beyond biological severity alone.^3^ EP is also strongly associated with worse cancer patient experience, again independent of stage.^4^

EP has been characterized in a small number of high-income countries other than the United States, using linked cancer registry and hospital administration data to conduct population-based analyses of EP prevalence, predictors, and prognosis. Among previous studies, two merit specific mention: in 2012, foundational work in England established EP as a marker of excess mortality across the first year after diagnosis, with the largest absolute effects in the months immediately after diagnosis.^5^ In 2022, International Cancer Benchmarking Partnership collaborators demonstrated that substantial though variable proportions of patients with 8 common cancer sites are diagnosed through an EP in Australia, Canada, Denmark, New Zealand, Norway, and the UK, with consistent patterns of the predictors and prognostic implications of emergency diagnosis across the 14 jurisdictions studied.^6^ A small number of prior US studies have focused on individual cancers, particularly lung and colorectal cancers, or relied on hospital-based cohorts (ie, not using linked population-based cancer registry and hospital administration data). Most relevant US studies have defined EP as ED contact preceding the diagnosis of cancer, without distinguishing whether ED encounters led to an emergency hospital admission or a discharge, although there is appreciable variation in the characteristics of patients between these 2 groups.^7,8^

We previously reported that ED involvement in cancer diagnosis among Medicare beneficiaries ranges from 8% to 40% depending on tumor site, stage, and patient demographics, with higher rates among older and racially minoritized patients.^9^ Here, we quantify the association between EP and all-cause mortality at 30, 90, and 365 days for 16 common cancers in a population-based cohort of Medicare beneficiaries. We additionally differentiate prognosis by the care pathway after ED contact: hospitalization vs outpatient referral.^7^

## Methods

We analyzed SEER-Medicare data, which links 22 population-based cancer registries (~48% of the US population) to Medicare fee-for-service claims. The cohort included beneficiaries diagnosed 2008 to 2017 with one of 16 cancers (bladder, female breast, colon, esophagus, kidney, leukemia, liver, lung, non-Hodgkin lymphoma, myeloma, ovary, pancreas, prostate, rectum, stomach, or uterus). We excluded patients younger than 66, with prior cancer history, diagnosed with in situ/nonmalignant disease, or lacking continuous Medicare fee-for-service enrollment from 12 months before through 3 months after diagnosis or until death. The University of North Carolina IRB approved this study (exempt, #22 to 2998).

We defined the diagnosis index date using a validated algorithm identifying the earliest cancer-specific ICD code within ± 1 month of the SEER diagnosis month.^9–11^ For 87% of patients, this approximated the start of cancer care; for the remainder lacking a relevant code, the date was imputed as the 15th day of the diagnosis month. All-cause mortality was ascertained from Medicare enrollment files, which capture death regardless of continuous enrollment status, and was assessed at 30, 90, and 365 days after the index date. We analyzed mortality within discrete time windows: 0 to 30 days, 30 to 90 days (conditional on 30-day survival), and 90 to 365 days (conditional on 90-day survival). This approach reduces confounding by presentation severity, which strongly predicts both EP status and short-term mortality,^12,13^ and allows assessment of whether EP-associated risk attenuates over time.

EP was defined as an ED-associated claim (revenue codes 0450 to 0459) or hospitalization with emergency admission type within 30 days before the index date. We further classified EPs as “inpatient EP” if the index date occurred during an emergency hospitalization, or “outpatient EP” when the index date followed ED discharge. Emergency hospitalizations included those with ED revenue codes, emergency admission type, or an ED visit within 3 days before unplanned admission to capture emergency transfers. Patients without ED claims or emergency hospitalizations were classified as “non-EP.”^11^ This algorithm and its variation by cancer site, stage, and patient characteristics have been described in detail elsewhere.^7^

Covariates included age (5-year groups), sex, race/ethnicity (non-Hispanic White [NHW], non-Hispanic Black [NHB], Hispanic, American Indian or Alaskan Native [AIAN], disaggregated Asian American groups [East Asian, Southeast Asian, South Asian, Other Asian], Native Hawaiian or other Pacific Islander [NHOPI], mixed race, and other/unknown), tumor stage (localized, regional, metastatic/distant, unstaged), site-specific histology groupings, Charlson Comorbidity Index,^14,15^ Kim frailty index,^16^ marital status, Medicaid dual eligibility, diagnosis year, SEER region, census-tract socioeconomic status (quintiles), metropolitan county residence, and health-care utilization in the year before the index date including any cancer screening (breast, cervical, prostate, lung, or colorectal) and counts of ED visits, outpatient office visits, and inpatient stays.

Analyses were performed in SAS 9.4 and R 4.4.0. Descriptive statistics were stratified by EP status. Kaplan-Meier curves described survival by EP status, stage, and cancer site. Site-specific mortality risks were estimated using direct standardization to the age, sex, and stage distribution of each cancer-specific cohort, with risk differences (RDs) calculated using non-EP as the reference. Poisson regression with robust standard errors^17^ estimated fully adjusted risk ratios (RRs) for each EP category. Statistical significance was defined as 2-sided *P*<.05. The supplementary methods section describes model fitting with functional form and variable selection. Final adjustment set included age, sex, stage, histology, comorbidity, frailty, prediagnostic health-care utilization, SEER region, census-tract socioeconomic status, metropolitan county residence, and diagnosis year. We used both direct standardization and regression modeling for complementary purposes: direct standardization provides interpretable site-specific absolute risk estimates adjusted for key surveillance factors (age, sex, stage); adjusted RRs quantify the relative increase in mortality risk after controlling for extensive additional covariates.

## Results

The study population included 929 378 patients (mean age 76 years, 51% male). Most were NHW (82%), followed by NHB (8%), Hispanic (6%), and Asian American (4%), and fewer than 1% were NHOPI or AIAN. Eighty-five percent resided in metropolitan counties, and 94% had an office visit in the year before diagnosis. Overall, 28% were diagnosed with cancer through an emergency route: 22% inpatient and 6% outpatient. Inpatient EP proportions varied widely by cancer type, from 5% for breast and prostate to >35% for stomach, colon, ovarian, and pancreatic cancers. Among sites with the lowest overall EP prevalence (female breast, prostate, uterus), 40% to 50% were outpatient EPs, but for most sites, inpatient EPs predominated, accounting for ≥80% of all EPs for myeloma, lung, rectum, liver, pancreas, leukemia, stomach, and colon. Compared with non-EPs, EPs were older (especially inpatient EPs, 45% aged 80+ years vs 24% outpatient EP vs 12% non-EP), more often female (52% vs 48% non-EP), frailer (18% very frail vs 7% non-EP), and more often diagnosed with metastatic/distant stage disease (39% vs 15% non-EP). EPs also had different prediagnostic health-care utilization patterns: 10% of EPs had no consultation office visits in the year before diagnosis compared with 4% of non-EPs, and EPs were less likely to have received any cancer screening in the prior year (20% vs 37% of non-EPs). Marital status and tumor grade had considerable missing data, which we retained in analyses using a missing indicator category rather than excluding observations. Among those with non-missing values, EPs were less common in patients with well-differentiated solid tumors (8% vs 14% non-EP) and more EPs were unmarried at diagnosis (55% vs 41% non-EP). Outpatient EPs were more likely to live in nonmetropolitan counties (23% vs 12% inpatient EPs) (Table 1).

Across all cancer sites, 1-year survival probability was highest for non-EPs (81%), lower for outpatient EPs (60%), and lowest for inpatient EPs (36%). One-year survival was substantially higher for localized stage cancers: 95% (non-EP), 85% (outpatient EP), and 63% (inpatient EP); compared with late-stage cancers, where survival dropped to 41%, 30%, and 18% (Table 2). Kaplan-Meier curves (Figures 1, 2; Tables 2, S1) showed survival probability decreased most rapidly among inpatient EPs, followed by outpatient EPs, with non-EPs surviving longest. Survival differences by EP status varied across sites, with clear separation for some cancers (liver, lung, esophagus, leukemia, lymphoma, rectum) and less distinction between inpatient and outpatient subgroups for others (stomach, colon, myeloma, breast, ovarian, uterine, bladder, kidney, prostate).

Standardized absolute risks and RDs are shown in Tables S2 and S3 and Figure 3. Across all follow-up intervals and cancer sites, standardized mortality risk was highest for inpatient EPs and lowest for non-EPs. In the first 30 days, comparing EP groups with non-EPs, RDs exceeded 20% for liver cancer and leukemia in the inpatient route and were 15% to 20% for pancreatic, esophageal, lung, ovarian cancers, and lymphoma. The outpatient route also conferred higher 30-day risk (RDs 6% to 8%) for these sites. For 90-day and 1-year mortality, these RDs were attenuated, but death risk remained ≥20% higher for leukemia and myeloma diagnosed via the inpatient route compared with non-EPs even at 1 year.

Adjustment for additional variables attenuated the association between EP and mortality (Table S5), yet even after adjustment, most estimates remained significantly elevated. The impact of adjustment varied by cancer site. For 30-day mortality, attenuation was most pronounced for breast cancer and hematological malignancies, where fully adjusted RRs were roughly half the minimally adjusted estimates. In contrast, adjustment had less impact for pancreatic, esophageal, stomach, and lung cancers, where covariates could not explain elevated risk and the EP-associated mortality risk persisted with minimal change (Figure 4, Table S4).

After adjustment (Figure 5), 30-day mortality risks were approximately 6-fold higher for inpatient EPs with lymphoma and breast cancer, and about 5-fold higher for myeloma, prostate, uterus, and bladder cancers. Among those surviving the first month, EP status continued to predict higher relative death risk within 90 days and at 1 year. RRs for 1-year mortality (conditional on surviving at least 90 days) for inpatient EPs (vs non-EPs) were significantly elevated for all cancer sites (overall RR = 1.51, 95% CI = 1.49 to 1.52), and highest for lymphoma (RR = 2.07, 95% CI = 1.97 to 2.18), myeloma (RR = 1.85, 95% CI = 1.73 to 1.99), leukemia (RR = 1.72, 95% CI = 1.64 to 1.81), prostate (RR = 1.74, 95% CI = 1.63 to 1.86), and bladder (RR = 1.57, 95% CI = 1.49 to 1.65). Lung, colon, and rectal cancers also showed elevated 1-year mortality associated with EP, although with smaller associations (rectum: RR = 1.44, 95% CI = 1.34 to 1.54; colon: RR = 1.34, 95% CI = 1.29 to 1.39; lung: RR = 1.26, 95% CI = 1.24 to 1.28). Notably, rectal cancer patients had higher EP-associated mortality risk than colon cancer patients across all time periods. Among outpatient EPs, 1-year RRs were lower in magnitude than for inpatient EPs (overall RR = 1.28, 95% CI = 1.26 to 1.30) but remained significantly elevated for all tumor sites except kidney and ovarian cancers, and they were highest for prostate (RR = 1.50, 95% CI = 1.38 to 1.62), lymphoma (RR = 1.39, 95% CI = 1.28 to 1.51), myeloma (RR = 1.28, 95% CI = 1.14 to 1.44), and leukemia (RR = 1.28, 95% CI = 1.17 to 1.41) (Figure 5; Table S5).

## Discussion

In this large population-based cohort of older adults diagnosed with 16 common cancers, we quantified the prevalence of EP and its association with short-term, all-cause mortality using both directly standardized absolute risks and multivariable-adjusted risk ratios.^18^ We found that EPs accounted for 28% of diagnoses, with notable variation by site, age, stage, frailty, and social factors. We also observed differences between inpatient and outpatient EP subgroups. Across all cancer sites, EPs had substantially higher death risks at 30 days, 90 days, and 1 year compared with those diagnosed through routine pathways, even after adjustment for stage, histology, comorbidity, frailty, and health-care utilization. Inpatient EPs consistently experienced the poorest survival, underscoring the severity of urgent hospital-based diagnoses.

Our findings address a gap in US population-based evidence on EP and extend the international evidence base by separating inpatient and outpatient EP as prognostically distinct subgroups. The 2022 International Cancer Benchmarking Partnership (ICBP) examined the frequency and prognostic implications for esophagogastric, colorectal, liver, pancreas, lung, and ovarian cancer in 14 jurisdictions across 6 high-income countries (2012 to 2017), showing that EP accounts for a large proportion of cases (24% to 43%) and is associated with higher short-term mortality, with patterns mirroring our findings.^6^ In Ontario, Canada (2014 to 2021), Grewal et al. reported elevated all-cause mortality among those with recent ED use compared with those without, with estimates similar to ours.^19^ Domestically, in the Veteran Affairs (VA) system (2016 to 2020), Kapadia and colleagues reported that lung and colorectal cancer EPs had approximately 80% higher adjusted odds of 1-year mortality.^8^ Khalaf et al. found 28% of VA colorectal cancers (2017 to 2021) were diagnosed emergently, with these patients experiencing lower odds of receiving treatment and 70% higher mortality risk.^20^ Another VA study demonstrated a nearly 2-fold increase in mortality for pancreatic cancer EPs (2007 to 2019).^21^ A recent population-based study conducted in rural Minnesota and Wisconsin reported higher cancer-specific mortality among adults with an ED visit compared with matched controls without ED contact (over a median follow-up of 6.3 years); however, this study did not distinguish ED visits that would be considered emergency presentations as defined here.^22^

Several factors are likely to contribute to the excess short-term mortality observed among EPs. First, patients presenting through the ED are more likely to have advanced or complicated disease, including advanced-stage tumors located in anatomically challenging sites or presenting with obstruction, perforation, or bleeding.^23^ A further gradient of risk was observed between EPs who were admitted and those who were discharged and were subsequently diagnosed as outpatients.^7^ Emergency presentation scenarios often require urgent interventions that limit options for optimal staging and curative treatment. EPs are less likely to receive guideline-concordant therapy, including surgical resection and multimodal treatment,^24^ and are more likely to experience perioperative complications^25^ and higher postoperative mortality.^26–28^ Histopathological types more common among EPs are also associated with poorer prognosis.^29,30^ Patients who are older, have greater functional decline,^31^ or have preferences favoring less aggressive care^32^ may be both more likely to present emergently and less likely to pursue curative treatment, contributing to observed mortality differences independent of the diagnostic pathway itself.^33^ Beyond disease severity, the context of initial treatment, especially emergency surgery, likely contributes to poorer outcomes.^34^ Urgent or unplanned surgical interventions are performed under suboptimal conditions,^35^ with higher risk of complications;^36–38^ inpatient mortality after emergency surgery is substantial and may partly explain the markedly elevated 30-day mortality for certain tumor sites.^37^ However, the relative contribution of these factors likely varies by cancer site.

Although prior US research on EP focused heavily on lung and colorectal cancers, our study shows EP was most strongly associated with excess short-term mortality for other sites—particularly lymphoma, myeloma, breast, prostate, and bladder cancers. These consistently ranked among the highest adjusted risk ratios across 30-day, 90-day, and 1-year follow-up, whereas lung and colorectal cancers did not. Notably, the impact of multivariable adjustment varied substantially by cancer site. For breast cancer and hematological malignancies, adjustment for comorbidity, frailty, and health-care utilization attenuated risk ratios by roughly half, suggesting that patient-level factors explain a substantial portion of the EP-mortality association. In contrast, adjustment had minimal impact for pancreatic, esophageal, stomach, and lung cancers, where EP-associated mortality risk persisted largely unchanged—suggesting that for these cancers, EP itself (or its downstream consequences) contributes to worse outcomes beyond measured confounders. For breast and prostate cancers, the rarity of EP (~5% inpatient) suggests most patients are diagnosed through routine pathways; those who present emergently may have experienced significant diagnostic delays or access barriers, resulting in complications that preclude optimal treatment.^39–41^ For hematological malignancies, EPs often involve life-threatening complications that increase immediate mortality and impair chemotherapy tolerance.^42–44^ In contrast, lung and colorectal cancers often present with advanced disease regardless of diagnostic route, and EP may facilitate timely curative intervention, partially offsetting excess risk.

Beyond the presentation severity, additional factors likely contribute to persistent survival disadvantage among EPs. Some patients may delay seeking care despite early symptoms due to fear, stigma, limited health literacy, or access barriers, allowing cancer to progress before diagnosis. Others may experience missed diagnostic opportunities related to provider- or health-care system factors, such as symptoms misattributed to other conditions^45^ or inadequate follow-up of abnormal results.^46,47^ New cancer symptoms may receive less attention due to competing demands of managing chronic comorbidities.^48^ Additionally, some patients, particularly those with limited primary care access, may use the ED as their primary source of routine care.^49^ For those individuals, an ED visit leading to cancer diagnosis may not represent a true emergency.^50–52^

Beyond its individual-level prognostic significance, EP may serve as a population-level indicator of diagnostic system performance, including primary care access and preventive screening quality. Internationally, EPs are widely reported as a standard cancer diagnostic performance metric and incorporated into prognostic scoring systems to stratify short-term mortality risk at diagnosis.^30,53^ Recent US policy developments signal growing recognition of this measurement gap; the US Agency for Healthcare Research and Quality (AHRQ) has proposed EP as a draft quality indicator for cancer diagnosis, currently specified for lung and colorectal cancers only; our findings across 16 cancer sites provide foundational evidence that could inform broader specification.^54^ Although claims data capture utilization rather than structural access to health care, our results are consistent with this framing: 10% of EPs had no consultation office visits in the year before diagnosis compared with 4% of non-EPs, and outpatient EPs were disproportionately represented among patients residing outside metropolitan counties. Companion work using the same cohort further showed that EPs had less prediagnostic nonemergency health-care use for cancer-relevant symptoms than non-EPs, suggesting reduced engagement with primary care for symptom evaluation in the year before diagnosis.^7^

A distinct clinical concern involves the downstream care pathway for patients with cancer identified in the ED. Emergency departments are not structured to coordinate oncology workup, and patients discharged with suspected malignancy enter a care pathway in which no single clinician or team reliably owns diagnostic coordination. This creates risks of delayed tissue diagnosis, inconsistent staging, and loss to follow-up, particularly when referral requires tissue confirmation that the ED cannot provide. Our finding that outpatient EPs had prediagnostic primary care engagement comparable to non-EPs suggests that missed diagnostic opportunities, rather than disconnection from routine care, may contribute to emergency presentation in this subgroup. The clinical implications of different EP care pathways, specifically whether patients with suspected malignancy identified in the ED should be routinely admitted for expedited diagnostic workup vs managed through outpatient referral, remain unclear and warrant further investigation.

Our findings reinforce the importance of EP as a strong and independent prognostic marker. However, routine measurement of emergency presentation remains challenging in the United States due to limited linkage of cancer registration with clinical and claims data, care delivery fragmentation, and variability in how ED visits are captured across health systems.^55^ Our study demonstrates the feasibility of identifying EP status in administrative data and highlights the need to distinguish inpatient from outpatient EPs, because these groups differ in characteristics, care pathways, and prognosis. Incorporating EP classification into survival analyses, risk prediction tools, quality improvement programs, and cancer surveillance could help target efforts to reduce preventable EPs and support more equitable diagnostic care.^56^ For patients who present emergently, recognizing EP as a strong adverse prognostic marker should inform clinical decision-making, including shared decision-making about treatment intensity, early integration of palliative care services, and advance care planning conversations, particularly for older adults with high comorbidity burden.

This study has limitations. First, we examined all-cause rather than cancer-specific survival. Although some deaths were from noncancer causes, we adjusted for comorbidity and frailty to account for differences in baseline morbidity burden, and all-cause mortality is clinically relevant because it captures the full impact of emergency presentation, including treatment-related complications and competing risks. Second, although we adjusted for detailed demographic, clinical, and prediagnostic health-care utilization factors, residual confounding is possible. In particular, unmeasured factors such as functional status, patient preferences regarding treatment intensity, and baseline health not fully captured by claims-based indices may independently influence both likelihood of emergency presentation and treatment decisions. Different presenting symptoms are also known to be associated with variation in diagnostic intervals and prognosis. The introduction of public health symptom awareness campaigns and interventions (such as the production of clinical guidelines) to increase the proportion of patients with new symptoms who are referred for urgent specialist investigation for suspected cancer have led to a reduction in the fraction of cancer patients diagnosed through an emergency presentation in England.^57^ A limitation of our study is that we had no information about the symptoms at presentation, nor the speed and type of subsequent investigations. Third, EP status was identified using claims within a fixed time window, which may misclassify patients if ED encounters were not reimbursed, bundled with other services, occurred outside the classification window, or were incidental and unrelated to subsequent cancer diagnosis, though we expect minimal differential misclassification. Fourth, we did not examine cancer-directed treatment as a potential mediator of the EP-mortality association. Prior Veterans Affairs analyses have shown that EPs are less likely to receive cancer-directed treatment,^8^ and treatment differences likely explain a portion of the observed mortality gap. Fifth, our 30-day classification window for outpatient EPs may underestimate true EP burden if diagnostic workup extends beyond this timeframe. Finally, our results may not generalize to patients younger than 65, uninsured populations, or those enrolled in Medicare Advantage plans.

In summary, this population-based study demonstrates that EP is common across cancers in US adults aged older than 65 and is consistently associated with substantially higher short-term mortality, even after accounting for stage and other tumor and patient factors. These findings highlight the need for systematic monitoring of EPs as a quality indicator and the importance of reducing preventable diagnostic delays.^1,58^ Targeted efforts to improve public symptom awareness, expand primary care access, and strengthen diagnostic coordination may help decrease cancers diagnosed as emergencies and improve survival. Future research should refine approaches to identify EP in cancer registries and real-world data, evaluate its utility in risk stratification, and inform policies aimed at equitable access to high-quality diagnostic pathways.

## Supplementary Material

Supplementary tables

Supplementary methods

Supplementary material

Supplementary material is available at *JNCI: Journal of the National Cancer Institute* online.

## Figures and Tables

**Figure 1.**
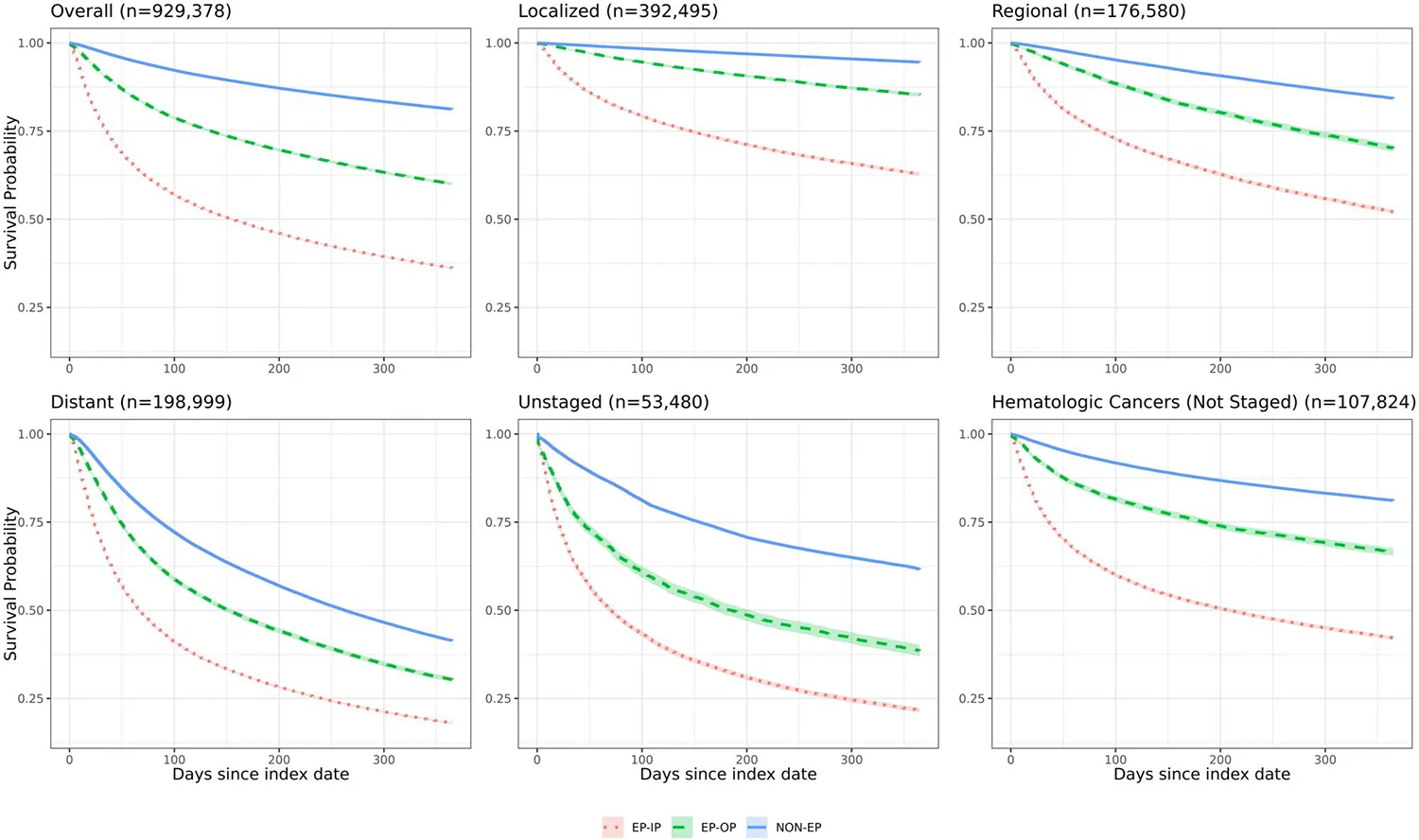
One-year survival by emergency presentation (EP) status, overall and stratified by tumor stage. Kaplan-Meier survival curves for inpatient EP (EP-IP, red dotted), outpatient EP (EP-OP, green dashed), and non-EP (blue solid) groups. Panels show overall survival (*n*=929 378) and survival stratified by stage: localized (*n*=392 495), regional (*n* = 176 580), distant (*n* = 198 999), unstaged solid tumors (*n* = 53 480), and hematological cancers (*n* = 107 824). Across all stage groups, inpatient EPs had the lowest survival probability, with the steepest decline occurring in the first 90 days after diagnosis. Outpatient EPs showed intermediate survival, whereas non-EPs had the highest survival throughout follow-up. Survival differences by EP status were most pronounced for distant-stage disease, where 1-year survival was approximately 18% for inpatient EPs, 30% for outpatient EPs, and 41% for non-EPs. Even among localized-stage cancers, inpatient EPs experienced notably lower survival (63%) compared with outpatient EPs (85%) and non-EPs (95%).

**Figure 2.**
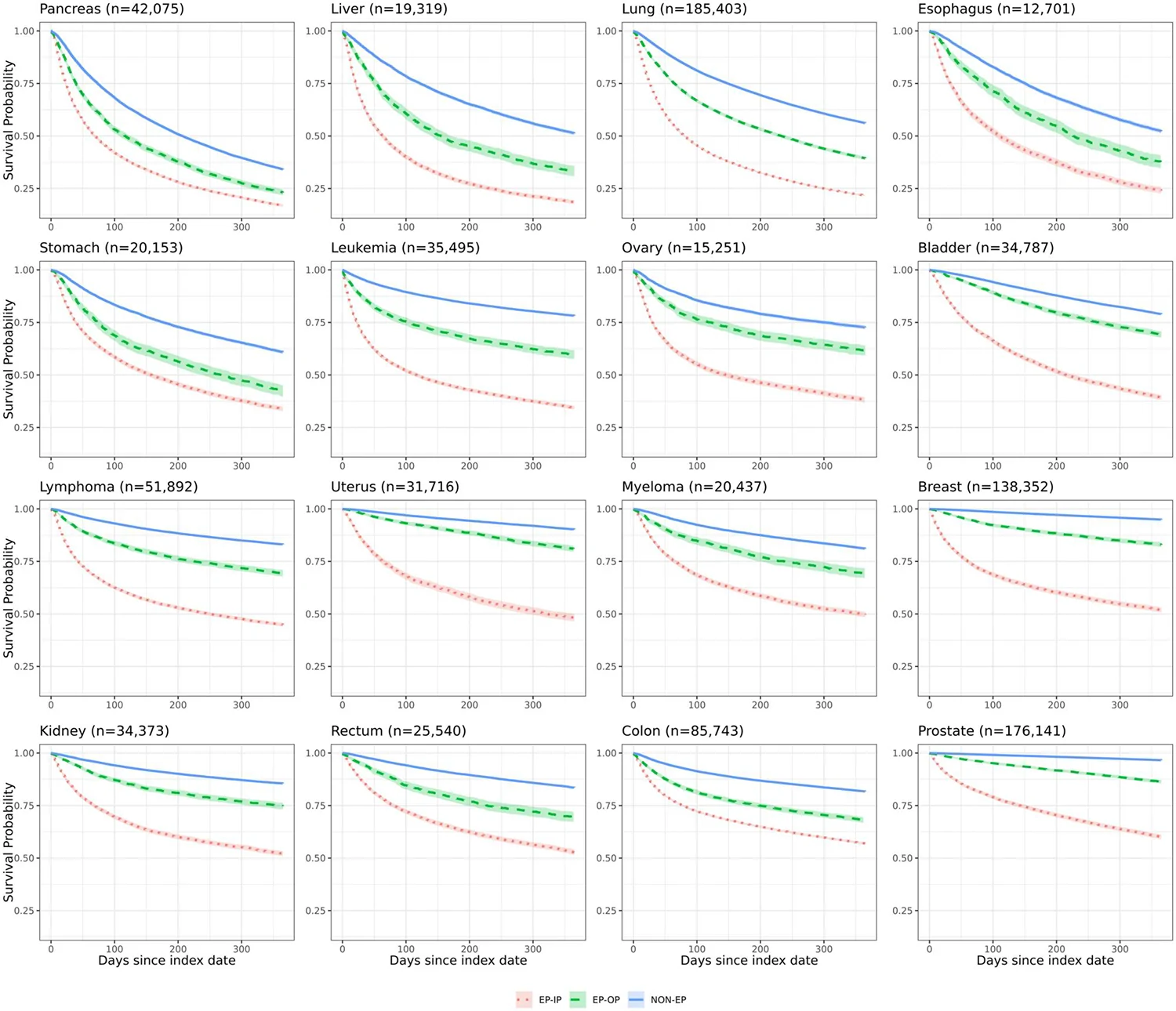
One-year survival by emergency presentation (EP) status, stratified by cancer site. Kaplan-Meier survival curves for inpatient EP (EP-IP, red dotted), outpatient EP (EP-OP, green dashed), and non-EP (blue solid) groups across 16 cancer sites. Panels are ordered by 1-year survival among inpatient EPs, from lowest (pancreas) to highest (prostate). For all cancer sites, inpatient EPs had the lowest survival, with the steepest declines in the first 90 days. Pancreatic cancer showed the poorest overall survival regardless of EP status, reflecting its aggressive biology. Clear and persistent separation across all 3 EP groups was evident for liver, lung, esophagus, leukemia, lymphoma, and rectal cancers. For stomach and colon cancers, EP was associated with worse survival, but distinctions between inpatient and outpatient subgroups were less pronounced. For myeloma, breast, ovarian, uterine, bladder, kidney, and prostate cancers, inpatient EP conveyed a marked survival disadvantage, whereas outpatient EP survival more closely resembled non-EPs. Sample sizes for each cancer site are shown in the panel headers.

**Figure 3.**
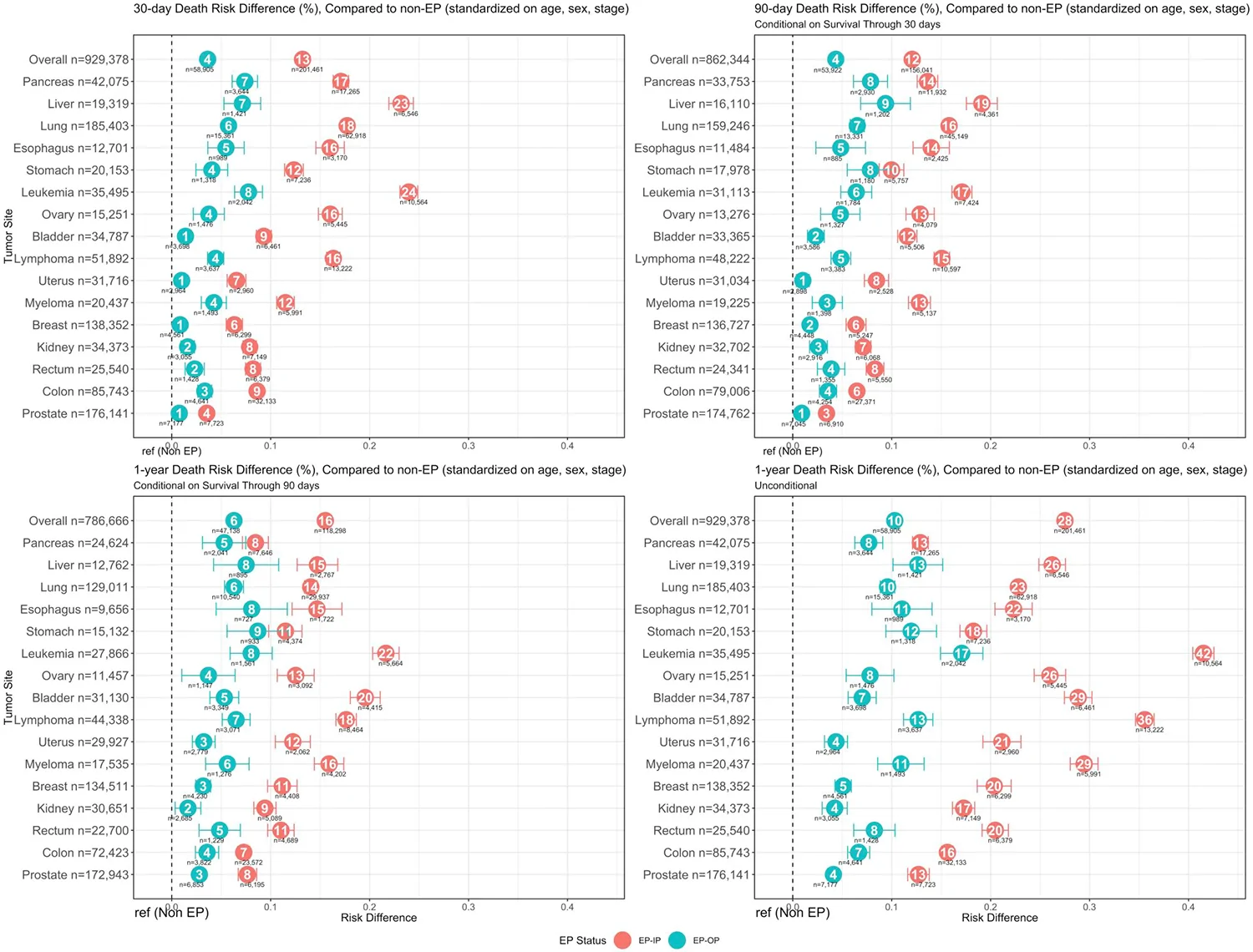
Standardized mortality risk differences comparing inpatient EP (EP-IP) and outpatient EP (EP-OP) with non-EP, by cancer site and follow-up period. Risk differences (percentage points) are standardized to the age, sex, and stage distribution of each cancer-specific cohort, with non-EP as the reference. Panels show 30-day mortality (top left), 90-day mortality conditional on surviving the first 30 days (top right), 1-year mortality conditional on surviving the first 90 days (bottom left), and unconditional 1-year mortality (bottom right). Point estimates are displayed with 95% confidence intervals; numbers inside points indicate the risk difference. For all cancer sites and time periods, inpatient EPs (red) had higher mortality risk than outpatient EPs (blue), and both EP groups had higher risk than non-EPs. The largest 30-day risk differences were observed for liver cancer and leukemia among inpatient EPs (>20 percentage points). Risk differences attenuated over time but remained elevated, particularly for hematological malignancies. Sample sizes vary across panels due to the conditional analytic approach: the 90-day conditional analysis excludes patients who died within 30 days, and the 1-year conditional analysis excludes those who died within 90 days, enabling assessment of whether EP-associated mortality risk persists beyond the acute diagnostic period.

**Figure 4.**
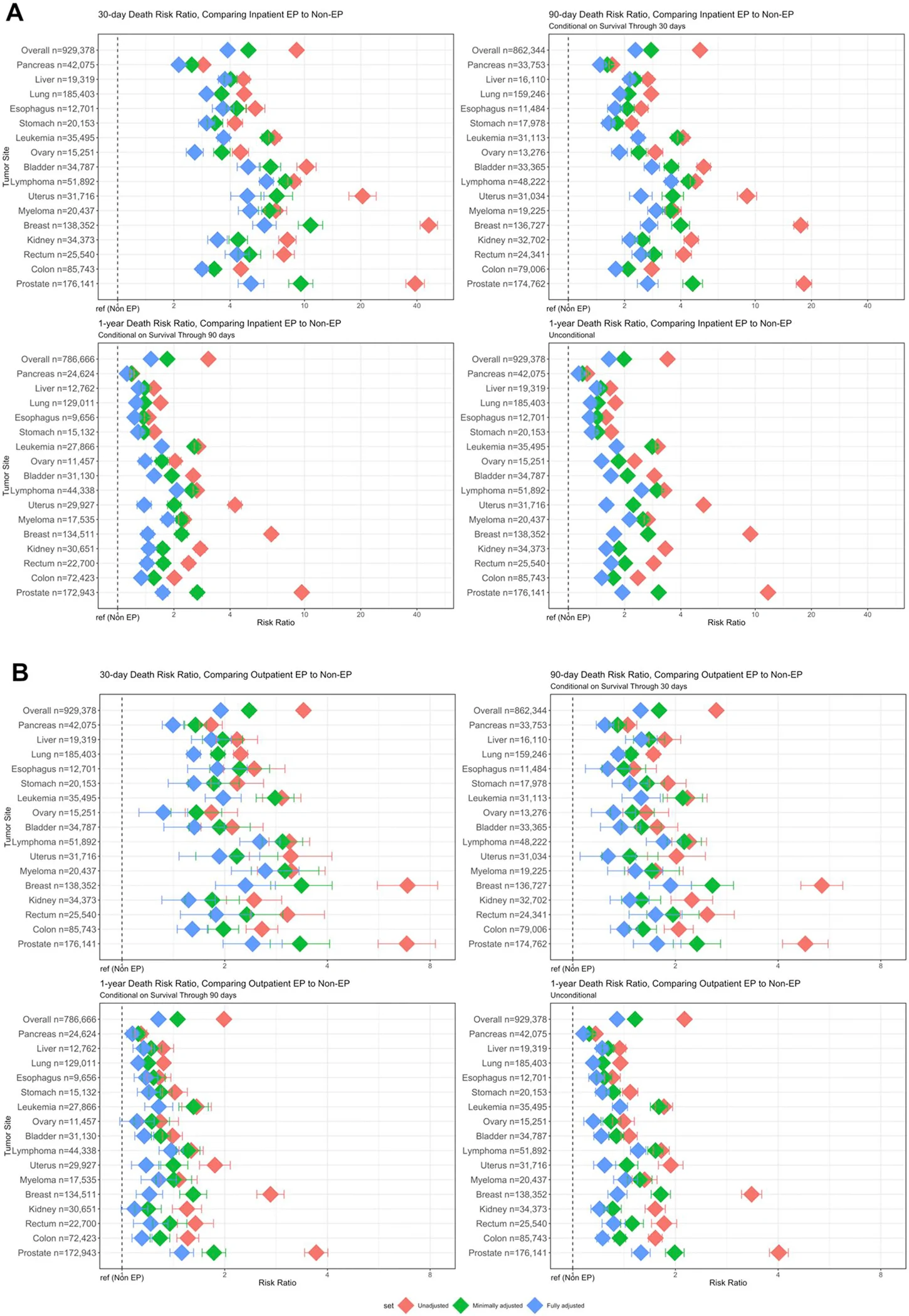
Impact of multivariable adjustment on risk ratios (RRs) comparing inpatient EP (EP-IP, Panel **A**) and outpatient EP (EP-OP, Panel **B**) with non-EP, by cancer site and follow-up period (on the log10 scale). RRs and 95% confidence intervals for conditional 1-year mortality (among 90-day survivors) are shown for unadjusted models (red), minimally adjusted models (green; adjusted for age, sex, and stage), and fully adjusted models (blue; additionally adjusted for histology, comorbidity, frailty, health-care utilization, and contextual characteristics including SEER region, census-tract socioeconomic status, metropolitan residence, and diagnosis year). Adjustment attenuated RRs for all cancer sites, but the magnitude of attenuation varied substantially. In Panel A, for inpatient EP, for breast cancer, hematological malignancies (leukemia, lymphoma, myeloma), and prostate cancer, adjustment in addition to the minimally adjusted set reduced 1-year unconditional RRs by roughly half, suggesting that patient-level factors explain a substantial portion of the EP-mortality association for these cancers. In addition to these cancer sites, cancers of bladder, uterus, kidney, colon, and rectum demonstrated great attenuation in 1-year unconditional RRs from unadjusted estimates. In contrast, adjustment had minimal impact on ovarian, liver, lung, esophagus, stomach, and pancreatic cancers, where EP-associated mortality risk persisted largely unchanged—suggesting that for these cancers, EP itself or its downstream consequences contributes to worse outcomes beyond measured confounders. Conditional 1-year mortality reflects death risk among patients who survived at least 90 days after diagnosis, reducing confounding by presentation severity and enabling assessment of whether EP-associated risk persists beyond the acute diagnostic period. Outpatient EP (Panel **B**) exhibits similar patterns in 1-year unconditional RRs as inpatient EP, except that the attenuations were less with additional adjustment among hematological malignancies (leukemia, lymphoma, myeloma).

**Figure 5.**
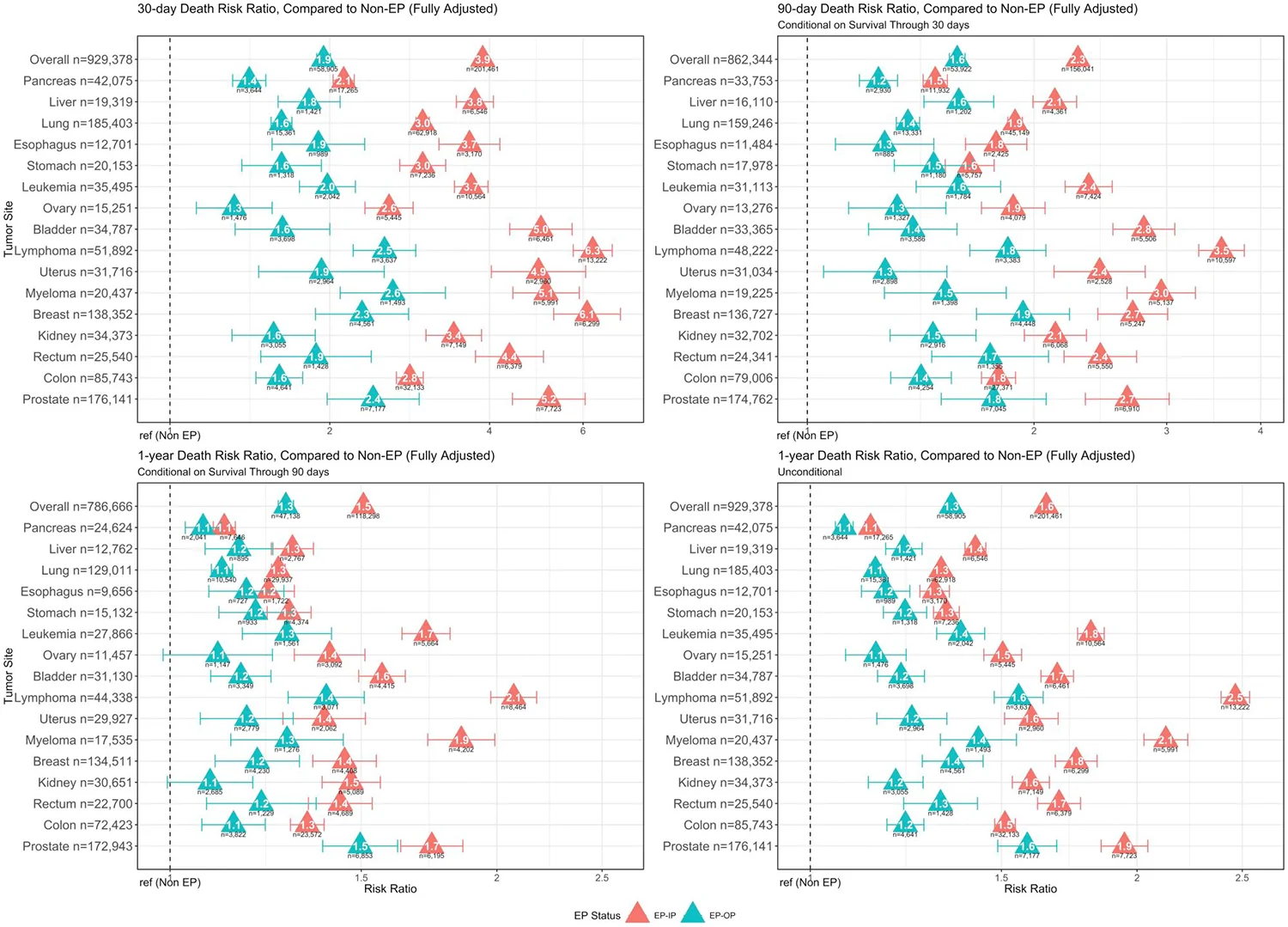
Adjusted risk ratios (RRs) comparing inpatient EP (EP-IP) and outpatient EP (EP-OP) with non-EP, by cancer site and follow-up period. RRs and 95% confidence intervals from modified Poisson regression models with robust standard errors, adjusted for age, sex, stage, histology, comorbidity, frailty, health-care utilization, and contextual characteristics (SEER region, census-tract socioeconomic status, metropolitan residence, diagnosis year). Panels show 30-day mortality (top left), 90-day mortality conditional on surviving the first 30 days (top right), 1-year mortality conditional on surviving the first 90 days (bottom left), and unconditional 1-year mortality (bottom right). Inpatient EPs (red triangles) had higher mortality risk than outpatient EPs (blue triangles) across all cancer sites and time periods; both EP groups had significantly elevated risk compared with non-EPs. The largest 30-day RRs were observed for lymphoma (6.3) and breast cancer (6.1) among inpatient EPs. EP-associated mortality risk attenuated over time but remained significantly elevated: among 90-day survivors, inpatient EPs had 51% higher 1-year mortality overall (RR = 1.51), with the highest RRs for lymphoma (2.07), myeloma (1.85), prostate (1.74), and leukemia (1.72). Outpatient EPs showed consistently intermediate risk, with 1-year RRs significantly elevated for all sites except kidney and ovarian cancers. Sample sizes vary across panels due to the conditional analytic approach, which excludes patients who died in earlier periods.

**Table 1.** Study population characteristics overall and by emergency presentation (EP) status.

Characteristic	All		Non-EP			Any EP			Inpatient EP			Outpatient EP		
	No.	%	No.	%		No.	%		No.	%		No.	%	
				Row	Col		Row	Col		Row	Col		Row	Col

All	929 378	100	669 012	72.0	100	260 366	28.0	100	201 461	21.7	100	58 905	6.3	100
Sex														
Male	471 716	50.8	347 526	73.7	51.9	124 190	26.3	47.7	94 676	20.1	47.0	29 514	6.3	50.1
Female	457 662	49.2	321 486	70.2	48.1	136 176	29.8	52.3	106 785	23.3	53.0	29 391	6.4	49.9
Age														
Mean (SD)	76.3	7.3	75.5	7.0		156	0.1		79.7	7.9		77.1	7.6	
66 to 69 years	198 932	21.4	157 676	79.3	23.6	41 256	20.7	15.8	30 019	15.1	14.9	11 237	5.6	19.1
70 to 74years	237 159	25.5	183 443	77.4	27.4	53 716	22.6	20.6	39 937	16.8	19.8	13 779	5.8	23.4
75 to 79years	196 869	21.2	143 898	73.1	21.5	52 971	26.9	20.3	40 578	20.6	20.1	12 393	6.3	21.0
80 to 84 years	151 510	16.3	101 554	67.0	15.2	49 956	33.0	19.2	39 658	26.2	19.7	10 298	6.8	17.5
85 to 89 years	97 129	10.5	57 967	59.7	8.7	39 162	40.3	15.0	31 863	32.8	15.8	7299	7.5	12.4
90 to 94 years	38 714	4.2	20 233	52.3	3.0	18 481	47.7	7.1	15 370	39.7	7.6	3111	8.0	5.3
95+ years	9065	1.0	4241	46.8	0.6	4824	53.2	1.9	4036	44.5	2.0	788	8.7	1.3
Race/ethnicity^a^														
Non-Hispanic White	753 533	81.8	548 984	72.9	83.0	204 549	27.1	78.8	156 949	20.8	78.1	47 600	6.3	80.8
Non-Hispanic Black	73 350	8.0	47 776	65.1	7.2	25 574	34.9	9.9	20 605	28.1	10.3	4969	6.8	8.4
Hispanic	51 971	5.6	35 110	67.6	5.3	16 861	32.4	6.5	13 241	25.5	6.6	3620	7.0	6.2
American Indian and Alaskan Native (AIAN)	3427	0.4	2385	69.6	0.4	1042	30.4	0.4	712	0.4	0.4	330	9.6	0.6
East Asian	19 295	2.1	13 784	71.4	2.1	5511	28.6	2.1	4628	24.0	2.3	883	4.6	1.5
Southeast Asian	10 608	1.2	6936	65.4	1.0	3672	34.6	1.4	3027	28.5	1.5	645	6.1	1.1
South Asian	3061	0.3	2286	74.7	0.3	775	25.3	0.3	606	19.8	0.3	169	5.5	0.3
Other Asian	4493	0.5	3480	77.5	0.5	1013	22.5	0.4	793	17.6	0.4	220	4.9	0.4
NHOPI	2347	0.3	1587	67.6	0.2	760	32.4	0.3	566	24.1	0.3	194	8.3	0.3
Other/Unknown	7293	–	6684	91.6	–	609	8.4	–	334	4.6	–	275	3.8	–
Charlson comorbidity score^b^														
0	401 931	44.2	319 766	79.6	48.8	82 165	20.4	32.5	57 882	14.4	29.8	24 283	6.0	41.6
1	225 533	24.8	164 994	73.2	25.2	60 539	26.8	23.9	45 508	20.2	23.4	15 031	6.7	25.7
2	122 957	13.5	82 377	67.0	12.6	40 580	33.0	16.0	32 306	26.3	16.6	8274	6.7	14.2
3+	157 904	17.4	88 269	55.9	13.5	69 635	44.1	27.5	58 841	37.3	30.2	10 794	6.8	18.5
Unknown^c^	21 053	–	13 606	64.6	–	7447	35.4	–	6924	32.9	–	523	2.5	–
Frailty index^d^														
Robust (<0.15)	99 368	10.7	49 978	50.3	7.5	49 390	49.7	19.0	40 920	41.2	20.3	8470	8.5	14.4
Prefrail (0.15–0.24)	30 540	3.3	11 666	38.2	1.7	18 874	61.8	7.2	16 516	54.1	8.2	2358	7.7	4.0
Mildly frail (0.25–0.34)	392 931	42.3	270 481	68.8	40.4	122 450	31.2	47.0	93 011	23.7	46.2	29 439	7.5	50.0
Moderate to severely frail (≥0.35)	406 539	43.7	336 887	82.9	50.4	69 652	17.1	26.8	51 014	12.5	25.3	18 638	4.6	31.6
Year of diagnosis														
2008 to 2009	203 875	21.9	149 499	73.3	22.3	54 376	26.7	20.9	43 528	21.4	21.6	10 848	5.3	18.4
2010 to 2011	191 945	20.7	139 004	72.4	20.8	52 941	27.6	20.3	41 880	21.8	20.8	11 061	5.8	18.8
2012 to 2014	268 663	28.9	191 516	71.3	28.6	77 147	28.7	29.6	59 276	22.1	29.4	17 871	6.7	30.3
2015 to 2017	264 895	28.5	188 993	71.3	28.2	75 902	28.7	29.2	56 777	21.4	28.2	19 125	7.2	32.5
Primary tumor site (ordered by any EP row %)														
Female breast	138 352	14.9	127 492	92.2	19.1	10 860	7.8	4.2	6299	4.6	3.1	4561	3.3	7.7
Prostate	176 141	19.0	161 241	91.5	24.1	14 900	8.5	5.7	7723	4.4	3.8	7177	4.1	12.2
Uterus	31 716	3.4	25 792	81.3	3.9	5924	18.7	2.3	2960	9.3	1.5	2964	9.3	5.0
Bladder	34 787	3.7	24 628	70.8	3.7	10 159	29.2	3.9	6461	18.6	3.2	3698	10.6	6.3
Kidney	34 373	3.7	24 169	70.3	3.6	10 204	29.7	3.9	7149	20.8	3.5	3055	8.9	5.2
Rectum	25 540	2.7	17 733	69.4	2.7	7807	30.6	3.0	6379	25.0	3.2	1428	5.6	2.4
Lymphoma	51 892	5.6	35 033	67.5	5.2	16 859	32.5	6.5	13 222	25.5	6.6	3637	7.0	6.2
Esophagus	12 701	1.4	8542	67.3	1.3	4159	32.7	1.6	3170	25.0	1.6	989	7.8	1.7
Leukemia	35 495	3.8	22 889	64.5	3.4	12 606	35.5	4.8	10 564	29.8	5.2	2042	5.8	3.5
Myeloma	20 437	2.2	12 953	63.4	1.9	7484	36.6	2.9	5991	29.3	3.0	1493	7.3	2.5
Liver	19 319	2.1	11 352	58.8	1.7	7967	41.2	3.1	6546	33.9	3.2	1421	7.4	2.4
Lung	185 403	19.9	107 124	57.8	16.0	78 279	42.2	30.1	62 918	33.9	31.2	15 361	8.3	26.1
Stomach	20 153	2.2	11 599	57.6	1.7	8554	42.4	3.3	7236	35.9	3.6	1318	6.5	2.2
Colon	85 743	9.2	48 969	57.1	7.3	36 774	42.9	14.1	32 133	37.5	15.9	4641	5.4	7.9
Ovary	15 251	1.6	8330	54.6	1.2	6921	45.4	2.7	5445	35.7	2.7	1476	9.7	2.5
Pancreas	42 075	4.5	21 166	50.3	3.2	20 909	49.7	8.0	17 265	41.0	8.6	3644	8.7	6.2
Tumor stage (SEER summary)														
Localized	392 495	42.2	338 408	86.2	50.6	54 087	13.8	20.8	35 317	9.0	17.5	18 770	4.8	31.9
Regional, direct extension only	73 542	7.9	52 487	71.4	7.8	21 055	28.6	8.1	16 137	21.9	8	4918	6.7	8.3
Regional, regional lymph nodes only	59 176	6.4	45 600	77.1	6.8	13 576	22.9	5.2	10 380	17.5	5.2	3196	5.4	5.4
Regional, direct extension and regional nodes	43 679	4.7	28 089	64.3	4.2	15 590	35.7	6.0	12 396	28.4	6.2	3194	7.3	5.4
Regional, NOS	183	0.0	110	60.1	0.0	73	39.9	0.0	60	32.8	0.0	13	7.1	0.0
Distant	198 999	21.4	97 803	49.1	14.6	101 196	50.9	38.9	83 141	41.8	41.3	18 055	9.1	30.7
Unstaged solid tumor	53 480	5.8	35 640	66.6	5.3	17 840	33.4	6.9	14 253	26.7	7.1	3587	6.7	6.1
Nonsolid tumor	107 824	11.6	70 875	65.7	10.6	36 949	34.3	14.2	29 777	27.6	14.8	7172	6.7	12.2
Tumor grade														
Well differentiated	83 785	12.5	72 849	86.9	13.9	10 936	13.1	7.6	7211	8.6	6.8	3725	4.4	9.8
Moderately differentiated	250 408	37.4	202 209	80.8	38.5	48 199	19.2	33.4	36 303	14.5	34.1	11896	4.8	31.4
Poorly differentiated	212 681	31.8	166 643	78.4	31.7	46 038	21.6	31.9	33 375	15.7	31.4	12 663	6.0	33.5
Undifferentiated; anaplastic	122 683	18.3	83 670	68.2	15.9	39 013	31.8	27.1	29 452	24.0	27.7	9561	7.8	25.3
Unknown^c^	259 821	–	143 641	55.3	–	116 180	44.7	–	95 120	36.6	–	21 060	8.1	–
Marital status														
Married/partnered	346 107	55.2	266 346	77.0	59.4	79 761	23.0	44.9	58 470	16.9	43.1	21 291	6.2	50.5
Single/divorced/widowed	280 408	44.8	182 389	65.0	40.6	98 019	35.0	55.1	77 146	27.5	56.9	20 873	7.4	49.5
Unknown^c^	302 863	–	220 277	72.7	–	82 586	27.3	–	65 845	21.7	–	16741	5.5	–
Medicaid eligibility^e^														
Any	148 484	16.0	87 084	58.6	13.0	61 400	41.4	23.6	49 466	33.3	24.6	11 934	8.0	20.3
None	780 894	84.0	581 928	74.5	87.0	198 966	25.5	76.4	151 995	19.5	75.4	46 971	6.0	79.7
SEER region^f^														
East	375 626	40.4	265 259	70.6	39.6	110 367	29.4	42.4	90 933	24.2	45.1	19 434	5.2	33.0
South	174 887	18.8	126 758	72.5	18.9	48 129	27.5	18.5	35 255	20.2	17.5	12 874	7.4	21.9
Midwest	80 711	8.7	56 824	70.4	8.5	23 887	29.6	9.2	17 745	22.0	8.8	6142	7.6	10.4
West	298 154	32.1	220 171	73.8	32.9	77 983	26.2	30.0	57 528	19.3	28.6	20 455	6.9	34.7
County geography^g^														
Metropolitan	791 586	85.2	569 738	72.0	85.2	221 848	28.0	85.2	176 864	22.3	87.8	44 984	5.7	76.4
Non-metropolitan/unknown^h^	137 792	14.8	99 274	72.0	14.8	38 518	28.0	14.8	24 597	17.9	12.2	13 921	10.1	23.6
Neighborhood SES (quintiles)^i^														
Lower	138 707	15.5	91 127	65.7	14.2	47 580	34.3	19.0	36 886	26.6	19.0	10 694	7.7	18.8
Lower middle	176 705	19.8	122 689	69.4	19.1	54 016	30.6	21.6	40 978	23.2	21.2	13 038	7.4	22.9
Middle	186 978	20.9	133 959	71.6	20.8	53 019	28.4	21.2	40 734	21.8	21.0	12 285	6.6	21.6
Upper middle	192 477	21.5	141 864	73.7	22.0	50 613	26.3	20.2	39 515	20.5	20.4	11 098	5.8	19.5
Upper	199 555	22.3	154 200	77.3	24.0	45 355	22.7	18.1	35 632	17.9	18.4	9723	4.9	17.1
Unknown^c^	34 956	–	25 173	72.0	–	9783	28.0	–	7716	22.1	–	2067	5.9	–
Count of ED visits in the prior year^j^														
0	629 997	67.8	493 032	78.3	73.7	136 96	21.7	52.6	105 649	16.8	52.4	31 316	5.0	53.2
1	176 349	19.0	115 212	65.3	17.2	61 137	34.7	23.5	47 160	26.7	23.4	13 977	7.9	23.7
2	65 128	7.0	36 041	55.3	5.4	29 087	44.7	11.2	22 798	35.0	11.3	6289	9.7	10.7
3+	57 904	6.2	24 727	42.7	3.7	33 177	57.3	12.7	25 854	44.6	12.8	7323	12.6	12.4
Count of consultation office visits in the prior year														
0	53 728	5.8	29 032	54.0	4.3	24 696	46.0	9.5	21 768	40.5	10.8	2928	5.4	5.0
1 to 4	195 281	21.0	135 731	69.5	20.3	59 550	30.5	22.9	47 360	24.3	23.5	12 190	6.2	20.7
5 to 8	242 129	26.1	182 035	75.2	27.2	60 094	24.8	23.1	46 022	19.0	22.8	14 072	5.8	23.9
9 to 12	178 629	19.2	133 828	74.9	20.0	44 801	25.1	17.2	33 385	18.7	16.6	11 416	6.4	19.4
13+	259 611	27.9	188 386	72.6	28.2	71 225	27.4	27.4	52 926	20.4	26.3	18 299	7.0	31.1
Count of inpatient stays in the prior year^j^														
0	773 968	83.3	562 891	72.7	84.1	211 07	27.3	81.1	164 140	21.2	81.5	46 937	6.1	79.7
1	106 036	11.4	76 800	72.4	11.5	29 236	27.6	11.2	21 209	20.0	10.5	8027	7.6	13.6
2	30 778	3.3	19 766	64.2	3.0	11 012	35.8	4.2	8563	27.8	4.3	2449	8.0	4.2
3+	18 596	2.0	9555	51.4	1.4	9041	48.6	3.5	7549	40.6	3.7	1492	8.0	2.5
Screening status in the prior year														
No	631 143	67.9	421 611	66.8	63.0	209 53	33.2	80.5	166 309	26.4	82.6	43 223	6.8	73.4
Yes	298 235	32.1	247 401	83.0	37.0	50 834	17.0	19.5	35 152	11.8	17.4	15 682	5.3	26.6

a Hispanic ethnicity includes patients with White, Black, “other,” or unknown race. American Indian and Alaska Native (AIAN) includes patients who are American Indian, Aleutian, Alaskan Native, or Eskimo. East Asian includes patients with Chinese, Korean, or Japanese ethnicity. Southeast Asian includes patients with Kampuchean, Laotian, Hmong, Thai, Filipino, or Vietnamese ethnicity. South Asian includes patients with Indian or Pakistani ethnicity. Native Hawaiian and Other Pacific Islander (NHOPI) includes patients who are Polynesian, Hawaiian, Samoan, Tahitian, Tongan, Micronesian, Guamanian, Chamorro, Melanesian, Fiji Islander, New Guinean, or Pacific Islander, NOS ethnicity.

b Modified Charlson comorbidity index based on National Cancer Institute macros that summarize inpatient and outpatient claims in the 365 days before diagnosis. Patients with no claims in the lookback period were classified as unknown.

c Unknown categories not included in percentage calculations.

d Claims-based frailty index as published by Kim et al. (2018) based on all claims in the 365 days before diagnosis.

e Eligible patients are qualified Medicare beneficiaries (QMBs), specified low-income Medicare beneficiaries (SLMBs), qualified disabled working individuals (QDWIs), and qualifying individuals (QIs). Full eligibility refers to patients enrolled in these programs who are also eligible for Medicaid coverage including prescription drugs.

f Based on US Census Bureau-designated regions: East: Connecticut, New Jersey, New York, Massachusetts. South: Georgia, Kentucky, and Louisiana. Midwest: metropolitan Detroit and Iowa. West: New Mexico, Utah, Idaho, California, Seattle and Puget Sound, and Hawaii.

g Based on Rural-Urban Continuum Codes. Counties that are metropolitan: RUCC 1 to 3; nonmetropolitan: RUCC 4 to 9.

h Categories were collapsed due to sparse counts for unknown values.

i Neighborhood SES assigned based on data from the American Community Survey describing the census tract of patient address at the time of diagnosis as published by Yost et al. (2001). It is a composite variable based on 7 tract-level attributes including median household income, median house value, median gross rent, percent of population living below 150% poverty, education index, percent working class, and percent unemployed.

j We subtract 1 from count of ED visits for both EP groups (inpatient and outpatient EP) and 1 from number of inpatient stays for inpatient EP group.

**Table 2.** Kaplan-Meier survival probability estimates and number at risk at 30, 90, 180, and 365 days by emergency presentation (EP) status, overall and by tumor stage.

		EP-IP		EP-OP		NON-EP	
Stage	Time (Days)	No.^a^	Survival probability (95% CI)	No.^a^	Survival probability (95% CI)	No.^a^	Survival probability (95% CI)

Overall	0	201 461	1.00 (1.00 to 1.00)	58 905	1.00 (1.00 to 1.00)	669 012	1.00 (1.00 to 1.00)
	30	157 093	0.78 (0.77 to 0.78)	54 065	0.92 (0.91 to 0.92)	652 976	0.98 (0.98 to 0.989)
	90	118 682	0.59 (0.59 to 0.59)	47 224	0.80 (0.80 to 0.80)	621 674	0.93 (0.92 to 0.93)
	180	96 107	0.48 (0.47 to 0.48)	41 923	0.71 (0.71 to 0.72)	589 396	0.88 (0.88 to 0.88)
	365	72 966	0.36 (0.36 to 0.36)	35 382	0.60 (0.60 to 0.60)	543 408	0.81 (0.81 to 0.81)
Distant	0	83 141	1.00 (1.00 to 1.00)	18 055	1.00 (1.00 to 1.00)	97 803	1.00 (1.00 to 1.00)
	30	58 096	0.69 (0.69 to 0.70)	15 244	0.84 (0.83 to 0.85)	89 458	0.91 (0.91 to 0.91)
	90	36 209	0.43 (0.43 to 0.44)	11 092	0.61 (0.60 to 0.62)	72 765	0.74 (0.74 to 0.74)
	180	25 080	0.30 (0.30 to 0.30)	8394	0.46 (0.46 to 0.47)	58 289	0.60 (0.59 to 0.60)
	365	15 037	0.18 (0.18 to 0.18)	5480	0.30 (0.30 to 0.31)	40 544	0.41 (0.41 to 0.42)
Regional	0	26 517	1.00 (1.00 to 1.00)	8114	1.00 (1.00 to 1.00)	98 087	1.00 (1.00 to 1.00)
	30	23 133	0.87 (0.87 to 0.87)	7814	0.96 (0.96 to 0.97)	96 890	0.99 (0.99 to 0.99)
	90	19 720	0.74 (0.74 to 0.75)	7267	0.90 (0.89 to 0.90)	93 900	0.96 (0.96 to 0.96)
	180	17 097	0.64 (0.64 to 0.65)	6619	0.82 (0.81 to 0.82)	89 769	0.92 (0.91 to 0.92)
	365	13 831	0.52 (0.52 to 0.53)	5699	0.70 (0.69 to 0.71)	82 710	0.84 (0.84 to 0.85)
Localized	0	35 317	1.00 (1.00 to 1.00)	18 770	1.00 (1.00 to 1.00)	338 408	1.00 (1.00 to 1.00)
	30	31 933	0.90 (0.90 to 0.91)	18 440	0.98 (0.98 to 0.98)	336 857	1.00 (1.00 to 1.00)
	90	28 433	0.80 (0.80 to 0.81)	17 848	0.95 (0.95 to 0.95)	333 519	0.99 (0.99 to 0.99)
	180	25 627	0.73 (0.72 to 0.73)	17 149	0.91 (0.91 to 0.92)	328 952	0.97 (0.97 to 0.97)
	365	22 197	0.63 (0.62 to 0.63)	16 023	0.85 (0.85 to 0.86)	319 997	0.95 (0.95 to 0.95)
Unstaged	0	14 253	1.00 (1.00 to 1.00)	3587	1.00 (1.00 to 1.00)	35 640	1.00 (1.00 to 1.00)
	30	9613	0.67 (0.66 to 0.68)	2869	0.79 (0.78 to 0.81)	33 162	0.93 (0.93 to 0.93)
	90	6461	0.45 (0.44 to 0.46)	2259	0.63 (0.61 to 0.64)	29 561	0.83 (0.82 to 0.83)
	180	4685	0.33 (0.32 to 0.34)	1806	0.50 (0.49 to 0.52)	25 889	0.73 (0.72 to 0.73)
	365	3086	0.22 (0.21 to 0.22)	1383	0.39 (0.37 to 0.40)	21 994	0.62 (0.61 to 0.62)
Hematological cancers (not staged)	0	29 777	1.00 (1.00 to 1.00)	7172	1.00 (1.00 to 1.00)	70 875	1.00 (1.00 to 1.00)
	30	23 293	0.78 (0.77 to 0.78)	6581	0.92 (0.91 to 0.92)	68 897	0.97 (0.97 to 0.97)
	90	18 378	0.62 (0.61 to 0.62)	5916	0.82 (0.82 to 0.83)	65 546	0.92 (0.92 to 0.93)
	180	15 477	0.52 (0.51 to 0.53)	5404	0.75 (0.74 to 0.76)	62 130	0.88 (0.87 to 0.88)
	365	12 560	0.42 (0.42 to 0.43)	4776	0.67 (0.66 to 0.68)	57 536	0.81 (0.81 to 0.81)

a This represents number of people at risk at each time point. Participants were followed for at least 1year, so there was no censoring in the data, and number of death events could be calculated based on the difference in number of people at risk between any 2 time points.

## Data Availability

The data underlying this article cannot be shared due to Institutional Review Board constraints around patient privacy. SEER-Medicare data can be accessed through an application to the National Cancer Institute with a SEER-Medicare Data Use Agreement and Institutional Review Board approval. Analytic code used in this analysis will be made available online.
