# Supplementary material for: Mortality after emergency presentation: evidence from a cohort of 929 378 Medicare beneficiaries with common cancers diagnosed 2008–2017
Source: J Natl Cancer Inst. Author manuscript; Available in PMC 2026 Oct 7. (PMC13645340; doi:10.1093/jnci/djag236)
Supplement: Supplementary file 1 [file NIHMS2209820-supplement-Supplementary_methods.docx]

**SUPPLEMENTARY METHODS**

We developed multivariable models to estimate the association between emergency presentation (EP) status and short-term mortality while adjusting for potential confounders. Our approach involved three steps: (1) determining optimal functional forms for continuous covariates, (2) selecting covariates using a forward selection strategy based on change-in-estimate criteria, and (3) specifying the final adjustment set. Models were fit separately for each of the 16 cancer sites. The directed acyclic graph (DAG) at the end of this section illustrates the assumed causal relationships between EP (exposure), short-term mortality (outcome), and covariates.

1. Functional form decisions. For the following continuous covariates, different functional forms were considered in univariate analyses for the unconditional one-year outcome, and the one that achieved the lowest value of Quasi-likelihood Information Criterion (QIC) was used for variable selection.
   a. Age: included as continuous linear.
      i. The forms considered were: 5-year grouping, continuous (linear), and quadratic (parabola).
      ii. Univariate model with continuous linear age achieved the lowest QIC for all site-stratified model, except for Leukemia, in which quadratic age yields a slightly lower QIC than linear age.
   b. Kim Frailty index: included in decile form (by tumor sites) based on empirical distribution.
      i. The forms considered were: tertiles, quartiles, quintiles, and deciles by tumor site; continuous linear, quadratic, and cubic; categorical [Robust (<0.15), Prefrail (0.15-0.24), Mildly frail (0.25-0.34), Moderate-to-severely frail (≥0.35)].
      ii. Models with frailty in deciles perform consistently better (across cancer site) than tertiles, quartiles, and quintiles;
      iii. Models with quadratic term perform consistently better (across cancer site) than models with just linear form or with cubic term;
      iv. The performance of models with quadratic term and deciles are very similar, so we choose deciles for better interpretability.
   c. Count of ED visits in the past year: included with linear and quadratic term.
      i. The Emergency Presentation groups (EP-IP and EP-OP) would have at least 1 ED visit in the [-30,0] time window. To avoid confounding with the definition of EP, we subtract 1 from this variable for both EP groups.
      ii. The forms considered were: categorical (0, 1, 2, 3+), binary (0, 1+), continuous linear, and continuous quadratic.
      iii. Because this variable is highly right-skewed (with 75% quantile at 0 and 90% quantile at 1), we do not consider the functional forms based on the empirical distribution.
      iv. Models with ED visit counts with quadratic term perform consistently better (across cancer site) than the other forms.
   d. Number of inpatient stays in the past year: categorized as 0, 1, 2, 3+
      i. The EP-IP group would have at least 1 inpatient stay in the [-30,0] time window. To avoid confounding with the definition of EP-IP, we subtract 1 from this variable for the EP-IP group.
      ii. The forms considered were: categorical (0, 1, 2, 3+), binary (0, 1+), continuous linear, and continuous quadratic.
      iii. Because this variable is highly right-skewed (with 75% quantile at 0 and 90% quantile at 1), we do not consider the functional forms based on the empirical distribution.
      iv. The stratified models with categorical inpatient stays and with quadratic term perform better than the other two forms consistently.
      v. Categorical form performs better than the quadratic form in 12 out of 16 stratified models.
   e. Number of outpatient office visits in the past year: included in decile form (by tumor sites) based on empirical distribution.
      i. The forms considered were: tertiles, quartiles, quintiles, and deciles by tumor site; continuous linear, and quadratic; categorical (0, 1-4, 5-8, 9-12, 13+).
      ii. Models with outpatient office visits in decile perform better than the categorical form in 15 out of 16 stratified model, and consistently better than all the other functional forms.
2. Variable selection strategies. Covariates in the model were selected using a forward strategy: adjustment sets were retained if their inclusion changed the EP estimate by >5% for the unconditional one-year outcome in any strata by tumor site.
   a. Sex & Age
   b. Stage
   c. Histology
   d. Health status indicators, including Charlson comorbidity index (categorized as 0, 1, 2, 3+) and Kim Frailty index (in deciles by tumor sites), based on claims in the 365 days prior to diagnosis.
   e. Healthcare utilization variables (in the prior year, i.e. no overlap with survival period), including indicator for screening status, count of ED visits (with linear and quadratic terms), number of inpatient stays (categorized as 0, 1, 2, 3+), and number of outpatient office visits (in deciles by tumor sites) in the past year, based on claims in the 365 days prior to diagnosis.
   f. Individual-level characteristics, including partnership status, Medicaid eligibility, and race/ethnicity of patients.
   g. Contextual characteristics, including SEER region, Yost composite score for neighborhood SES based on tract-level attributes (in quintiles by counties), county-level metropolitan status of residency, and year of diagnoses.
3. Final adjustment set. Based on the results in Supplementary Table S5, the inclusion of individual-level characteristics did not change the estimate by 5% or above. So, the final adjustment included age, sex, stage, histology, comorbidity, frailty, healthcare utilization in the 365 days prior to the index date (any cancer screening, outpatient office visits, ED visits, inpatient stays), SEER region, census-tract socioeconomic status, metropolitan residence, and diagnosis year.
4. Directed acyclic graph
