# Supplementary material for: Mortality after emergency presentation: evidence from a cohort of 929 378 Medicare beneficiaries with common cancers diagnosed 2008–2017
Source: J Natl Cancer Inst. Author manuscript; Available in PMC 2026 Oct 7. (PMC13645340; doi:10.1093/jnci/djag236)
Supplement: Supplementary file 2 [file NIHMS2209820-supplement-Supplementary_tables.docx]

Table S1. Kaplan-Meier survival probability estimates and number at risk at 30, 90, 180, and 365 days by emergency presentation (EP) status and tumor site.

|  |  | **EP-IP** | | **EP-OP** | | **NON-EP** | |
| --- | --- | --- | --- | --- | --- | --- | --- |
| **Tumor site** | **Time (Days)** | **N^a^** | **Survival probability** | **N^a^** | **Time** | **N^a^** | **Survival probability** |
| Pancreas | 0 | 17,265 | 1.000 (1.000-1.000) | 3,644 | 1.000 (1.000-1.000) | 21,166 | 1.000 (1.000-1.000) |
|  | 30 | 12,059 | 0.691 (0.684-0.698) | 2,956 | 0.804 (0.791-0.817) | 18,983 | 0.893 (0.888-0.897) |
|  | 90 | 7,684 | 0.443 (0.436-0.450) | 2,051 | 0.560 (0.544-0.576) | 14,988 | 0.706 (0.700-0.712) |
|  | 180 | 5,252 | 0.303 (0.296-0.310) | 1,472 | 0.403 (0.387-0.419) | 11,400 | 0.537 (0.530-0.544) |
|  | 365 | 2,929 | 0.169 (0.164-0.175) | 844 | 0.231 (0.218-0.245) | 7,233 | 0.341 (0.334-0.347) |
| Liver | 0 | 6,546 | 1.000 (1.000-1.000) | 1,421 | 1.000 (1.000-1.000) | 11,352 | 1.000 (1.000-1.000) |
|  | 30 | 4,400 | 0.666 (0.655-0.678) | 1,213 | 0.846 (0.827-0.865) | 10,566 | 0.929 (0.924-0.934) |
|  | 90 | 2,786 | 0.423 (0.411-0.435) | 897 | 0.630 (0.605-0.655) | 9,119 | 0.802 (0.794-0.809) |
|  | 180 | 1,903 | 0.289 (0.279-0.301) | 663 | 0.465 (0.440-0.492) | 7,660 | 0.674 (0.665-0.683) |
|  | 365 | 1,214 | 0.185 (0.176-0.194) | 472 | 0.331 (0.308-0.357) | 5,834 | 0.513 (0.504-0.522) |
| Lung | 0 | 62,918 | 1.000 (1.000-1.000) | 15,361 | 1.000 (1.000-1.000) | 107,124 | 1.000 (1.000-1.000) |
|  | 30 | 45,572 | 0.718 (0.714-0.721) | 13,377 | 0.868 (0.863-0.873) | 101,008 | 0.941 (0.939-0.942) |
|  | 90 | 30,097 | 0.476 (0.472-0.480) | 10,574 | 0.686 (0.679-0.694) | 88,708 | 0.826 (0.824-0.829) |
|  | 180 | 21,741 | 0.344 (0.341-0.348) | 8,517 | 0.553 (0.545-0.561) | 76,668 | 0.715 (0.712-0.717) |
|  | 365 | 13,663 | 0.217 (0.213-0.220) | 6,064 | 0.394 (0.387-0.402) | 60,184 | 0.561 (0.558-0.564) |
| Esophagus | 0 | 3,170 | 1.000 (1.000-1.000) | 989 | 1.000 (1.000-1.000) | 8,542 | 1.000 (1.000-1.000) |
|  | 30 | 2,439 | 0.765 (0.750-0.780) | 892 | 0.895 (0.876-0.914) | 8,193 | 0.957 (0.953-0.961) |
|  | 90 | 1,729 | 0.543 (0.526-0.561) | 731 | 0.735 (0.708-0.763) | 7,227 | 0.844 (0.836-0.851) |
|  | 180 | 1,263 | 0.398 (0.381-0.415) | 569 | 0.572 (0.542-0.604) | 6,040 | 0.706 (0.696-0.716) |
|  | 365 | 766 | 0.241 (0.226-0.256) | 375 | 0.376 (0.347-0.408) | 4,475 | 0.523 (0.512-0.534) |
| Stomach | 0 | 7,236 | 1.000 (1.000-1.000) | 1,318 | 1.000 (1.000-1.000) | 11,599 | 1.000 (1.000-1.000) |
|  | 30 | 5,808 | 0.796 (0.786-0.805) | 1,184 | 0.895 (0.879-0.912) | 11,066 | 0.952 (0.948-0.956) |
|  | 90 | 4,388 | 0.604 (0.593-0.616) | 935 | 0.708 (0.684-0.733) | 9,839 | 0.847 (0.841-0.854) |
|  | 180 | 3,451 | 0.476 (0.464-0.487) | 773 | 0.586 (0.560-0.613) | 8,674 | 0.747 (0.739-0.755) |
|  | 365 | 2,451 | 0.338 (0.327-0.349) | 559 | 0.423 (0.398-0.451) | 7,066 | 0.609 (0.600-0.618) |
| Leukemia | 0 | 10,564 | 1.000 (1.000-1.000) | 2,042 | 1.000 (1.000-1.000) | 22,889 | 1.000 (1.000-1.000) |
|  | 30 | 7,487 | 0.703 (0.694-0.712) | 1,790 | 0.874 (0.859-0.888) | 21,928 | 0.957 (0.954-0.960) |
|  | 90 | 5,684 | 0.536 (0.527-0.546) | 1,563 | 0.764 (0.746-0.783) | 20,660 | 0.902 (0.898-0.906) |
|  | 180 | 4,673 | 0.442 (0.432-0.451) | 1,405 | 0.687 (0.667-0.707) | 19,452 | 0.850 (0.845-0.854) |
|  | 365 | 3,638 | 0.344 (0.335-0.353) | 1,219 | 0.596 (0.576-0.618) | 17,905 | 0.782 (0.777-0.787) |
| Ovary | 0 | 5,445 | 1.000 (1.000-1.000) | 1,476 | 1.000 (1.000-1.000) | 8,330 | 1.000 (1.000-1.000) |
|  | 30 | 4,116 | 0.749 (0.738-0.761) | 1,332 | 0.899 (0.884-0.915) | 7,882 | 0.945 (0.940-0.950) |
|  | 90 | 3,108 | 0.568 (0.555-0.581) | 1,148 | 0.777 (0.756-0.799) | 7,224 | 0.867 (0.859-0.874) |
|  | 180 | 2,586 | 0.475 (0.461-0.488) | 1,034 | 0.700 (0.677-0.724) | 6,691 | 0.803 (0.794-0.811) |
|  | 365 | 2,079 | 0.381 (0.369-0.394) | 909 | 0.614 (0.590-0.640) | 6,054 | 0.726 (0.717-0.736) |
| Bladder | 0 | 6,461 | 1.000 (1.000-1.000) | 3,698 | 1.000 (1.000-1.000) | 24,628 | 1.000 (1.000-1.000) |
|  | 30 | 5,544 | 0.852 (0.844-0.861) | 3,588 | 0.970 (0.964-0.975) | 24,289 | 0.986 (0.984-0.987) |
|  | 90 | 4,426 | 0.683 (0.672-0.695) | 3,354 | 0.906 (0.896-0.915) | 23,385 | 0.949 (0.946-0.952) |
|  | 180 | 3,498 | 0.541 (0.529-0.553) | 3,011 | 0.814 (0.801-0.826) | 21,939 | 0.890 (0.886-0.894) |
|  | 365 | 2,542 | 0.392 (0.381-0.404) | 2,562 | 0.692 (0.677-0.707) | 19,454 | 0.790 (0.784-0.795) |
| Lymphoma | 0 | 13,222 | 1.000 (1.000-1.000) | 3,637 | 1.000 (1.000-1.000) | 35,033 | 1.000 (1.000-1.000) |
|  | 30 | 10,653 | 0.801 (0.795-0.808) | 3,392 | 0.930 (0.922-0.938) | 34,267 | 0.977 (0.976-0.979) |
|  | 90 | 8,484 | 0.640 (0.632-0.648) | 3,076 | 0.844 (0.833-0.856) | 32,821 | 0.936 (0.934-0.939) |
|  | 180 | 7,190 | 0.543 (0.535-0.552) | 2,825 | 0.776 (0.763-0.790) | 31,231 | 0.891 (0.888-0.894) |
|  | 365 | 5,943 | 0.449 (0.441-0.458) | 2,522 | 0.693 (0.678-0.708) | 29,120 | 0.831 (0.827-0.835) |
| Uterus | 0 | 2,960 | 1.000 (1.000-1.000) | 2,964 | 1.000 (1.000-1.000) | 25,792 | 1.000 (1.000-1.000) |
|  | 30 | 2,541 | 0.854 (0.841-0.867) | 2,901 | 0.978 (0.972-0.983) | 25,615 | 0.993 (0.992-0.994) |
|  | 90 | 2,070 | 0.697 (0.680-0.713) | 2,780 | 0.938 (0.929-0.946) | 25,092 | 0.973 (0.971-0.975) |
|  | 180 | 1,769 | 0.596 (0.579-0.614) | 2,651 | 0.894 (0.883-0.905) | 24,469 | 0.948 (0.946-0.951) |
|  | 365 | 1,428 | 0.482 (0.464-0.500) | 2,403 | 0.810 (0.796-0.825) | 23,275 | 0.902 (0.899-0.906) |
| Myeloma | 0 | 5,991 | 1.000 (1.000-1.000) | 1,493 | 1.000 (1.000-1.000) | 12,953 | 1.000 (1.000-1.000) |
|  | 30 | 5,153 | 0.857 (0.849-0.866) | 1,399 | 0.936 (0.924-0.949) | 12,702 | 0.980 (0.977-0.982) |
|  | 90 | 4,210 | 0.701 (0.690-0.713) | 1,277 | 0.855 (0.837-0.873) | 12,065 | 0.931 (0.926-0.935) |
|  | 180 | 3,614 | 0.602 (0.590-0.615) | 1,174 | 0.786 (0.765-0.807) | 11,447 | 0.883 (0.878-0.889) |
|  | 365 | 2,979 | 0.497 (0.485-0.510) | 1,036 | 0.693 (0.670-0.717) | 10,511 | 0.811 (0.804-0.818) |
| Breast | 0 | 6,299 | 1.000 (1.000-1.000) | 4,561 | 1.000 (1.000-1.000) | 127,492 | 1.000 (1.000-1.000) |
|  | 30 | 5,260 | 0.833 (0.824-0.842) | 4,452 | 0.975 (0.971-0.980) | 127,042 | 0.996 (0.996-0.997) |
|  | 90 | 4,412 | 0.700 (0.689-0.711) | 4,236 | 0.927 (0.920-0.935) | 125,896 | 0.987 (0.987-0.988) |
|  | 180 | 3,886 | 0.617 (0.605-0.629) | 4,060 | 0.889 (0.880-0.899) | 124,188 | 0.974 (0.973-0.975) |
|  | 365 | 3,273 | 0.519 (0.507-0.532) | 3,785 | 0.829 (0.819-0.840) | 121,007 | 0.949 (0.948-0.950) |
| Kidney | 0 | 7,149 | 1.000 (1.000-1.000) | 3,055 | 1.000 (1.000-1.000) | 24,169 | 1.000 (1.000-1.000) |
|  | 30 | 6,091 | 0.849 (0.841-0.857) | 2,920 | 0.955 (0.947-0.962) | 23,734 | 0.981 (0.980-0.983) |
|  | 90 | 5,107 | 0.712 (0.701-0.722) | 2,688 | 0.879 (0.867-0.891) | 22,892 | 0.947 (0.944-0.949) |
|  | 180 | 4,395 | 0.614 (0.603-0.626) | 2,496 | 0.816 (0.803-0.830) | 21,963 | 0.908 (0.905-0.912) |
|  | 365 | 3,734 | 0.522 (0.511-0.534) | 2,285 | 0.748 (0.732-0.763) | 20,678 | 0.855 (0.851-0.860) |
| Rectum | 0 | 6,379 | 1.000 (1.000-1.000) | 1,428 | 1.000 (1.000-1.000) | 17,733 | 1.000 (1.000-1.000) |
|  | 30 | 5,570 | 0.870 (0.862-0.878) | 1,356 | 0.949 (0.938-0.960) | 17,448 | 0.983 (0.981-0.985) |
|  | 90 | 4,702 | 0.735 (0.724-0.746) | 1,233 | 0.861 (0.843-0.879) | 16,793 | 0.946 (0.943-0.950) |
|  | 180 | 4,079 | 0.639 (0.627-0.650) | 1,119 | 0.783 (0.762-0.805) | 16,005 | 0.902 (0.898-0.907) |
|  | 365 | 3,372 | 0.528 (0.516-0.540) | 993 | 0.695 (0.671-0.719) | 14,814 | 0.835 (0.830-0.841) |
| Colon | 0 | 32,133 | 1.000 (1.000-1.000) | 4,641 | 1.000 (1.000-1.000) | 48,969 | 1.000 (1.000-1.000) |
|  | 30 | 27,473 | 0.852 (0.848-0.856) | 4,265 | 0.917 (0.909-0.925) | 47,437 | 0.968 (0.966-0.969) |
|  | 90 | 23,592 | 0.734 (0.729-0.738) | 3,824 | 0.824 (0.813-0.835) | 45,054 | 0.920 (0.917-0.922) |
|  | 180 | 21,249 | 0.661 (0.655-0.666) | 3,524 | 0.759 (0.747-0.771) | 42,894 | 0.876 (0.873-0.878) |
|  | 365 | 18,311 | 0.569 (0.564-0.575) | 3,524 | 0.759 (0.747-0.771) | 40,029 | 0.817 (0.814-0.821) |
| Prostate | 0 | 7,723 | 1.000 (1.000-1.000) | 7,177 | 1.000 (1.000-1.000) | 161,241 | 1.000 (1.000-1.000) |
|  | 30 | 6,927 | 0.895 (0.888-0.902) | 7,048 | 0.982 (0.979-0.985) | 160,816 | 0.997 (0.997-0.998) |
|  | 90 | 6,203 | 0.802 (0.793-0.811) | 6,857 | 0.955 (0.950-0.960) | 159,911 | 0.992 (0.991-0.992) |
|  | 180 | 5,558 | 0.719 (0.709-0.729) | 6,633 | 0.924 (0.918-0.930) | 158,675 | 0.984 (0.983-0.985) |
|  | 365 | 4,647 | 0.601 (0.590-0.612) | 6,201 | 0.863 (0.855-0.871) | 155,769 | 0.966 (0.965-0.967) |

a. This represents number of people at risk at each time point. Participants were followed for at least 1 year, so there were no censoring in the data, and number of death events could be calculated based on the difference in number of people at risk between any two time points.

Table S2. Crude and standardized mortality risks by emergency presentation (EP) status and tumor site for 30-day, 90-day (conditional on 30-day survival), 365-day (conditional on 90-day survival), and 365-day unconditional follow-up periods.

| **Period** | **Site** | **EP-IP** | | | | **EP-OP** | | | | **Non-EP** | | | |
| --- | --- | --- | --- | --- | --- | --- | --- | --- | --- | --- | --- | --- | --- |
|  |  | **Risk-C^a^** | **95% CI^b^** | **Risk-S^c^** | **95% CI^b^** | **Risk-C^c^** | **95% CI^b^** | **Risk-S^c^** | **95% CI^b^** | **Risk-C^a^** | **95% CI^b^** | **Risk-S^c^** | **95% CI^b^** |
| 30-day | Overall | 0.23 | (0.22-0.23) | 0.16 | (0.16-0.17) | 0.08 | (0.08-0.09) | 0.07 | (0.07-0.07) | 0.02 | (0.02-0.03) | 0.03 | (0.03-0.03) |
|  | Pancreas | 0.31 | (0.30-0.32) | 0.29 | (0.28-0.29) | 0.20 | (0.18-0.21) | 0.19 | (0.18-0.20) | 0.11 | (0.10-0.11) | 0.12 | (0.11-0.12) |
|  | Liver | 0.33 | (0.32-0.35) | 0.31 | (0.30-0.32) | 0.15 | (0.14-0.17) | 0.15 | (0.13-0.17) | 0.07 | (0.07-0.08) | 0.08 | (0.07-0.08) |
|  | Lung | 0.28 | (0.28-0.29) | 0.25 | (0.24-0.25) | 0.13 | (0.13-0.14) | 0.13 | (0.12-0.13) | 0.06 | (0.06-0.06) | 0.07 | (0.07-0.07) |
|  | Esophagus | 0.24 | (0.22-0.25) | 0.21 | (0.19-0.22) | 0.11 | (0.09-0.12) | 0.10 | (0.09-0.12) | 0.04 | (0.04-0.05) | 0.05 | (0.04-0.05) |
|  | Stomach | 0.20 | (0.20-0.21) | 0.18 | (0.17-0.19) | 0.10 | (0.09-0.12) | 0.10 | (0.08-0.11) | 0.05 | (0.04-0.05) | 0.06 | (0.05-0.06) |
|  | Leukemia | 0.30 | (0.29-0.31) | 0.28 | (0.28-0.29) | 0.13 | (0.11-0.14) | 0.12 | (0.11-0.14) | 0.04 | (0.04-0.05) | 0.05 | (0.04-0.05) |
|  | Ovary | 0.25 | (0.24-0.26) | 0.22 | (0.21-0.23) | 0.10 | (0.09-0.12) | 0.10 | (0.09-0.12) | 0.06 | (0.05-0.06) | 0.06 | (0.06-0.07) |
|  | Bladder | 0.15 | (0.14-0.16) | 0.11 | (0.10-0.12) | 0.03 | (0.02-0.04) | 0.03 | (0.03-0.04) | 0.01 | (0.01-0.02) | 0.02 | (0.02-0.02) |
|  | Lymphoma | 0.20 | (0.19-0.21) | 0.19 | (0.18-0.19) | 0.07 | (0.06-0.08) | 0.07 | (0.06-0.08) | 0.02 | (0.02-0.02) | 0.02 | (0.02-0.03) |
|  | Uterus | 0.15 | (0.13-0.16) | 0.07 | (0.07-0.08) | 0.02 | (0.02-0.03) | 0.02 | (0.01-0.02) | 0.01 | (0.01-0.01) | 0.01 | (0.01-0.01) |
|  | Myeloma | 0.14 | (0.13-0.15) | 0.14 | (0.13-0.14) | 0.06 | (0.05-0.08) | 0.06 | (0.05-0.08) | 0.02 | (0.02-0.02) | 0.02 | (0.02-0.02) |
|  | Breast | 0.17 | (0.16-0.18) | 0.07 | (0.06-0.08) | 0.02 | (0.02-0.03) | 0.01 | (0.01-0.02) | 0.00 | (0.00-0.00) | 0.00 | (0.00-0.01) |
|  | Kidney | 0.15 | (0.14-0.16) | 0.10 | (0.10-0.11) | 0.05 | (0.04-0.05) | 0.04 | (0.03-0.05) | 0.02 | (0.02-0.02) | 0.02 | (0.02-0.03) |
|  | Rectum | 0.13 | (0.12-0.14) | 0.10 | (0.10-0.11) | 0.05 | (0.04-0.06) | 0.04 | (0.03-0.05) | 0.02 | (0.01-0.02) | 0.02 | (0.02-0.02) |
|  | Colon | 0.15 | (0.14-0.15) | 0.13 | (0.12-0.13) | 0.08 | (0.08-0.09) | 0.07 | (0.07-0.08) | 0.03 | (0.03-0.03) | 0.04 | (0.04-0.04) |
|  | Prostate | 0.11 | (0.10-0.11) | 0.04 | (0.03-0.04) | 0.02 | (0.02-0.02) | 0.01 | (0.01-0.01) | 0.00 | (0.00-0.00) | 0.00 | (0.00-0.00) |
| 90-day (conditional on survival through the first 30 days) | Overall | 0.24 | (0.24-0.24) | 0.19 | (0.18-0.19) | 0.13 | (0.12-0.13) | 0.11 | (0.11-0.11) | 0.05 | (0.05-0.05) | 0.07 | (0.06-0.07) |
|  | Pancreas | 0.36 | (0.35-0.37) | 0.37 | (0.36-0.38) | 0.30 | (0.29-0.32) | 0.31 | (0.30-0.33) | 0.21 | (0.20-0.22) | 0.24 | (0.23-0.24) |
|  | Liver | 0.37 | (0.35-0.38) | 0.35 | (0.34-0.36) | 0.26 | (0.23-0.28) | 0.25 | (0.23-0.28) | 0.14 | (0.13-0.14) | 0.16 | (0.15-0.17) |
|  | Lung | 0.34 | (0.33-0.34) | 0.30 | (0.30-0.31) | 0.21 | (0.20-0.22) | 0.21 | (0.20-0.22) | 0.12 | (0.12-0.12) | 0.15 | (0.14-0.15) |
|  | Esophagus | 0.29 | (0.27-0.31) | 0.27 | (0.25-0.29) | 0.18 | (0.15-0.20) | 0.18 | (0.15-0.20) | 0.12 | (0.11-0.13) | 0.13 | (0.12-0.14) |
|  | Stomach | 0.24 | (0.23-0.25) | 0.23 | (0.22-0.24) | 0.21 | (0.19-0.23) | 0.21 | (0.19-0.23) | 0.11 | (0.10-0.12) | 0.13 | (0.12-0.14) |
|  | Leukemia | 0.24 | (0.23-0.25) | 0.23 | (0.22-0.24) | 0.13 | (0.11-0.14) | 0.13 | (0.11-0.14) | 0.06 | (0.05-0.06) | 0.06 | (0.06-0.06) |
|  | Ovary | 0.24 | (0.23-0.26) | 0.23 | (0.22-0.24) | 0.14 | (0.12-0.15) | 0.15 | (0.13-0.17) | 0.08 | (0.08-0.09) | 0.10 | (0.09-0.11) |
|  | Bladder | 0.20 | (0.19-0.21) | 0.16 | (0.15-0.17) | 0.07 | (0.06-0.07) | 0.07 | (0.06-0.08) | 0.04 | (0.04-0.04) | 0.05 | (0.04-0.05) |
|  | Lymphoma | 0.20 | (0.19-0.21) | 0.20 | (0.19-0.20) | 0.09 | (0.08-0.10) | 0.09 | (0.08-0.10) | 0.04 | (0.04-0.04) | 0.04 | (0.04-0.05) |
|  | Uterus | 0.18 | (0.17-0.20) | 0.11 | (0.10-0.12) | 0.04 | (0.03-0.05) | 0.04 | (0.03-0.04) | 0.02 | (0.02-0.02) | 0.03 | (0.02-0.03) |
|  | Myeloma | 0.18 | (0.17-0.19) | 0.18 | (0.17-0.19) | 0.09 | (0.07-0.10) | 0.09 | (0.07-0.10) | 0.05 | (0.05-0.05) | 0.05 | (0.05-0.06) |
|  | Breast | 0.16 | (0.15-0.17) | 0.08 | (0.07-0.09) | 0.05 | (0.04-0.06) | 0.03 | (0.03-0.03) | 0.01 | (0.01-0.01) | 0.01 | (0.01-0.01) |
|  | Kidney | 0.16 | (0.15-0.17) | 0.12 | (0.11-0.13) | 0.08 | (0.07-0.09) | 0.08 | (0.07-0.08) | 0.04 | (0.03-0.04) | 0.05 | (0.05-0.05) |
|  | Rectum | 0.16 | (0.15-0.16) | 0.13 | (0.12-0.14) | 0.09 | (0.08-0.11) | 0.08 | (0.07-0.10) | 0.04 | (0.03-0.04) | 0.05 | (0.04-0.05) |
|  | Colon | 0.14 | (0.13-0.14) | 0.13 | (0.12-0.13) | 0.10 | (0.09-0.11) | 0.10 | (0.09-0.11) | 0.05 | (0.05-0.05) | 0.06 | (0.06-0.07) |
|  | Prostate | 0.10 | (0.10-0.11) | 0.04 | (0.04-0.05) | 0.03 | (0.02-0.03) | 0.02 | (0.01-0.02) | 0.01 | (0.01-0.01) | 0.01 | (0.01-0.01) |
| 365-day (conditional on survival through the first 90 days) | Overall | 0.38 | (0.38-0.39) | 0.33 | (0.33-0.33) | 0.25 | (0.25-0.25) | 0.24 | (0.23-0.24) | 0.13 | (0.12-0.13) | 0.18 | (0.17-0.18) |
|  | Pancreas | 0.62 | (0.61-0.63) | 0.66 | (0.65-0.67) | 0.59 | (0.57-0.61) | 0.63 | (0.61-0.65) | 0.52 | (0.51-0.53) | 0.58 | (0.57-0.59) |
|  | Liver | 0.56 | (0.54-0.58) | 0.57 | (0.55-0.58) | 0.47 | (0.44-0.51) | 0.49 | (0.46-0.52) | 0.36 | (0.35-0.37) | 0.42 | (0.41-0.43) |
|  | Lung | 0.54 | (0.54-0.55) | 0.53 | (0.53-0.54) | 0.43 | (0.42-0.43) | 0.45 | (0.44-0.46) | 0.32 | (0.32-0.32) | 0.39 | (0.39-0.39) |
|  | Esophagus | 0.56 | (0.53-0.58) | 0.56 | (0.54-0.58) | 0.49 | (0.45-0.52) | 0.49 | (0.46-0.53) | 0.38 | (0.37-0.39) | 0.41 | (0.40-0.42) |
|  | Stomach | 0.44 | (0.43-0.46) | 0.45 | (0.44-0.46) | 0.40 | (0.37-0.43) | 0.42 | (0.39-0.45) | 0.28 | (0.27-0.29) | 0.33 | (0.33-0.34) |
|  | Leukemia | 0.36 | (0.35-0.37) | 0.36 | (0.35-0.37) | 0.22 | (0.20-0.24) | 0.22 | (0.20-0.24) | 0.13 | (0.13-0.14) | 0.14 | (0.14-0.15) |
|  | Ovary | 0.33 | (0.31-0.35) | 0.33 | (0.31-0.35) | 0.21 | (0.19-0.23) | 0.24 | (0.22-0.27) | 0.16 | (0.15-0.17) | 0.20 | (0.19-0.21) |
|  | Bladder | 0.43 | (0.41-0.44) | 0.39 | (0.38-0.41) | 0.24 | (0.22-0.25) | 0.25 | (0.24-0.26) | 0.17 | (0.16-0.17) | 0.20 | (0.19-0.20) |
|  | Lymphoma | 0.30 | (0.29-0.31) | 0.30 | (0.29-0.31) | 0.18 | (0.17-0.19) | 0.19 | (0.17-0.20) | 0.11 | (0.11-0.12) | 0.12 | (0.12-0.12) |
|  | Uterus | 0.31 | (0.29-0.33) | 0.22 | (0.20-0.23) | 0.14 | (0.12-0.15) | 0.13 | (0.11-0.14) | 0.07 | (0.07-0.08) | 0.09 | (0.09-0.10) |
|  | Myeloma | 0.29 | (0.28-0.31) | 0.29 | (0.28-0.31) | 0.19 | (0.17-0.21) | 0.19 | (0.17-0.21) | 0.13 | (0.12-0.13) | 0.14 | (0.13-0.14) |
|  | Breast | 0.26 | (0.24-0.27) | 0.16 | (0.14-0.17) | 0.11 | (0.10-0.11) | 0.08 | (0.07-0.09) | 0.04 | (0.04-0.04) | 0.05 | (0.05-0.05) |
|  | Kidney | 0.27 | (0.25-0.28) | 0.23 | (0.22-0.24) | 0.15 | (0.14-0.16) | 0.16 | (0.14-0.17) | 0.10 | (0.09-0.10) | 0.14 | (0.13-0.14) |
|  | Rectum | 0.28 | (0.27-0.29) | 0.26 | (0.24-0.27) | 0.19 | (0.17-0.21) | 0.19 | (0.17-0.21) | 0.12 | (0.11-0.12) | 0.15 | (0.14-0.15) |
|  | Colon | 0.22 | (0.22-0.23) | 0.23 | (0.22-0.23) | 0.17 | (0.16-0.19) | 0.19 | (0.18-0.20) | 0.11 | (0.11-0.11) | 0.15 | (0.15-0.16) |
|  | Prostate | 0.25 | (0.24-0.26) | 0.11 | (0.10-0.12) | 0.10 | (0.09-0.10) | 0.06 | (0.06-0.07) | 0.03 | (0.03-0.03) | 0.03 | (0.03-0.03) |
| 365-day (unconditional) | Overall | 0.64 | (0.64-0.64) | 0.51 | (0.51-0.51) | 0.40 | (0.40-0.40) | 0.34 | (0.33-0.34) | 0.19 | (0.19-0.19) | 0.23 | (0.23-0.23) |
|  | Pancreas | 0.83 | (0.83-0.84) | 0.81 | (0.81-0.82) | 0.77 | (0.76-0.78) | 0.76 | (0.75-0.77) | 0.66 | (0.65-0.67) | 0.68 | (0.68-0.69) |
|  | Liver | 0.82 | (0.81-0.82) | 0.78 | (0.77-0.79) | 0.67 | (0.64-0.69) | 0.65 | (0.62-0.67) | 0.49 | (0.48-0.50) | 0.52 | (0.51-0.53) |
|  | Lung | 0.78 | (0.78-0.79) | 0.72 | (0.71-0.72) | 0.61 | (0.60-0.61) | 0.58 | (0.58-0.59) | 0.44 | (0.44-0.44) | 0.49 | (0.49-0.49) |
|  | Esophagus | 0.76 | (0.74-0.77) | 0.72 | (0.70-0.74) | 0.62 | (0.59-0.65) | 0.61 | (0.58-0.64) | 0.48 | (0.47-0.49) | 0.50 | (0.49-0.51) |
|  | Stomach | 0.66 | (0.65-0.67) | 0.61 | (0.60-0.62) | 0.58 | (0.55-0.60) | 0.55 | (0.52-0.57) | 0.39 | (0.38-0.40) | 0.43 | (0.42-0.44) |
|  | Leukemia | 0.66 | (0.65-0.67) | 0.64 | (0.63-0.65) | 0.40 | (0.38-0.42) | 0.40 | (0.38-0.42) | 0.22 | (0.21-0.22) | 0.23 | (0.22-0.23) |
|  | Ovary | 0.62 | (0.61-0.63) | 0.57 | (0.55-0.58) | 0.39 | (0.36-0.41) | 0.39 | (0.36-0.41) | 0.27 | (0.26-0.28) | 0.31 | (0.30-0.32) |
|  | Bladder | 0.61 | (0.60-0.62) | 0.52 | (0.51-0.54) | 0.31 | (0.29-0.32) | 0.30 | (0.29-0.32) | 0.21 | (0.21-0.22) | 0.23 | (0.23-0.24) |
|  | Lymphoma | 0.55 | (0.54-0.56) | 0.53 | (0.52-0.54) | 0.31 | (0.29-0.32) | 0.30 | (0.29-0.32) | 0.17 | (0.17-0.17) | 0.18 | (0.17-0.18) |
|  | Uterus | 0.52 | (0.50-0.54) | 0.33 | (0.31-0.35) | 0.19 | (0.18-0.20) | 0.16 | (0.15-0.17) | 0.10 | (0.09-0.10) | 0.12 | (0.11-0.12) |
|  | Myeloma | 0.50 | (0.49-0.52) | 0.49 | (0.48-0.50) | 0.31 | (0.28-0.33) | 0.30 | (0.28-0.33) | 0.19 | (0.18-0.20) | 0.19 | (0.19-0.20) |
|  | Breast | 0.48 | (0.47-0.49) | 0.26 | (0.25-0.28) | 0.17 | (0.16-0.18) | 0.11 | (0.10-0.12) | 0.05 | (0.05-0.05) | 0.06 | (0.06-0.06) |
|  | Kidney | 0.48 | (0.47-0.49) | 0.35 | (0.34-0.36) | 0.25 | (0.24-0.27) | 0.22 | (0.21-0.24) | 0.14 | (0.14-0.15) | 0.18 | (0.18-0.19) |
|  | Rectum | 0.47 | (0.46-0.48) | 0.40 | (0.38-0.41) | 0.31 | (0.28-0.33) | 0.27 | (0.25-0.29) | 0.16 | (0.16-0.17) | 0.19 | (0.19-0.20) |
|  | Colon | 0.43 | (0.43-0.44) | 0.38 | (0.37-0.38) | 0.32 | (0.31-0.33) | 0.29 | (0.27-0.30) | 0.18 | (0.18-0.19) | 0.22 | (0.22-0.22) |
|  | Prostate | 0.40 | (0.39-0.41) | 0.17 | (0.16-0.18) | 0.14 | (0.13-0.14) | 0.08 | (0.08-0.09) | 0.03 | (0.03-0.03) | 0.04 | (0.04-0.04) |

a. Crude risks of death.
b. 95% Confidence intervals were constructed using normal approximation.
c. Standardized risks of death were implemented through direct standardization based on the distribution of 5-year age group, sex, and stage by tumor sites in the total population.

Table S3. Standardized risk differences (RDs) comparing inpatient EP (EP-IP) and outpatient EP (EP-OP) with non-EP by tumor site and follow-up period. Risk differences are standardized to the age, sex, and stage distribution of each cancer-specific cohort. Follow-up periods include 30-day, 90-day (conditional on 30-day survival), 365-day (conditional on 90-day survival), and 365-day unconditional.

| **Period** | **Site** | **EP-IP** | | **EP-OP** | |
| --- | --- | --- | --- | --- | --- |
|  |  | **RDs^a^** | **95% CI^b^** | **RDs^a^** | **95% CI^b^** |
| 30-day | Overall | 0.132 | (0.130-0.134) | 0.036 | (0.034-0.038) |
|  | Pancreas | 0.171 | (0.163-0.179) | 0.074 | (0.061-0.087) |
|  | Liver | 0.232 | (0.219-0.244) | 0.071 | (0.053-0.090) |
|  | Lung | 0.177 | (0.174-0.181) | 0.057 | (0.052-0.063) |
|  | Esophagus | 0.160 | (0.146-0.174) | 0.055 | (0.036-0.073) |
|  | Stomach | 0.123 | (0.114-0.133) | 0.041 | (0.024-0.057) |
|  | Leukemia | 0.240 | (0.231-0.249) | 0.078 | (0.064-0.092) |
|  | Ovary | 0.160 | (0.148-0.172) | 0.037 | (0.022-0.053) |
|  | Bladder | 0.093 | (0.086-0.101) | 0.014 | (0.008-0.020) |
|  | Lymphoma | 0.163 | (0.157-0.170) | 0.044 | (0.036-0.053) |
|  | Uterus | 0.066 | (0.056-0.075) | 0.010 | (0.005-0.015) |
|  | Myeloma | 0.115 | (0.106-0.124) | 0.043 | (0.030-0.055) |
|  | Breast | 0.063 | (0.055-0.072) | 0.009 | (0.006-0.011) |
|  | Kidney | 0.079 | (0.072-0.086) | 0.016 | (0.009-0.023) |
|  | Rectum | 0.082 | (0.075-0.090) | 0.023 | (0.014-0.033) |
|  | Colon | 0.086 | (0.082-0.090) | 0.033 | (0.026-0.040) |
|  | Prostate | 0.035 | (0.030-0.040) | 0.008 | (0.005-0.010) |
| 90-day (conditional on survival through the first 30 days) | Overall | 0.121 | (0.119-0.123) | 0.043 | (0.041-0.046) |
|  | Pancreas | 0.136 | (0.126-0.147) | 0.079 | (0.061-0.096) |
|  | Liver | 0.191 | (0.175-0.207) | 0.094 | (0.069-0.119) |
|  | Lung | 0.158 | (0.153-0.163) | 0.065 | (0.058-0.072) |
|  | Esophagus | 0.140 | (0.122-0.158) | 0.049 | (0.023-0.074) |
|  | Stomach | 0.100 | (0.087-0.112) | 0.079 | (0.055-0.102) |
|  | Leukemia | 0.171 | (0.161-0.181) | 0.064 | (0.049-0.080) |
|  | Ovary | 0.129 | (0.114-0.143) | 0.048 | (0.028-0.068) |
|  | Bladder | 0.116 | (0.106-0.125) | 0.023 | (0.015-0.032) |
|  | Lymphoma | 0.151 | (0.143-0.158) | 0.049 | (0.039-0.059) |
|  | Uterus | 0.085 | (0.072-0.097) | 0.010 | (0.004-0.017) |
|  | Myeloma | 0.128 | (0.117-0.139) | 0.035 | (0.020-0.050) |
|  | Breast | 0.064 | (0.054-0.074) | 0.017 | (0.013-0.022) |
|  | Kidney | 0.071 | (0.063-0.079) | 0.026 | (0.017-0.035) |
|  | Rectum | 0.083 | (0.074-0.092) | 0.039 | (0.025-0.053) |
|  | Colon | 0.065 | (0.060-0.069) | 0.036 | (0.027-0.044) |
|  | Prostate | 0.034 | (0.029-0.039) | 0.009 | (0.006-0.012) |
| 365-day (conditional on survival through the first 90 days) | Overall | 0.155 | (0.152-0.158) | 0.063 | (0.059-0.067) |
|  | Pancreas | 0.085 | (0.071-0.098) | 0.053 | (0.031-0.075) |
|  | Liver | 0.147 | (0.127-0.168) | 0.075 | (0.042-0.108) |
|  | Lung | 0.141 | (0.134-0.147) | 0.063 | (0.053-0.073) |
|  | Esophagus | 0.147 | (0.122-0.172) | 0.081 | (0.045-0.117) |
|  | Stomach | 0.115 | (0.098-0.132) | 0.087 | (0.056-0.118) |
|  | Leukemia | 0.216 | (0.203-0.230) | 0.080 | (0.059-0.102) |
|  | Ovary | 0.125 | (0.107-0.144) | 0.037 | (0.010-0.064) |
|  | Bladder | 0.196 | (0.180-0.211) | 0.053 | (0.039-0.068) |
|  | Lymphoma | 0.176 | (0.166-0.187) | 0.065 | (0.051-0.079) |
|  | Uterus | 0.122 | (0.105-0.140) | 0.032 | (0.021-0.044) |
|  | Myeloma | 0.159 | (0.144-0.174) | 0.056 | (0.034-0.078) |
|  | Breast | 0.112 | (0.097-0.127) | 0.032 | (0.024-0.039) |
|  | Kidney | 0.094 | (0.083-0.105) | 0.016 | (0.003-0.029) |
|  | Rectum | 0.110 | (0.097-0.124) | 0.049 | (0.028-0.069) |
|  | Colon | 0.073 | (0.067-0.079) | 0.036 | (0.024-0.047) |
|  | Prostate | 0.076 | (0.067-0.086) | 0.028 | (0.023-0.033) |
| 365-day (unconditional) | Overall | 0.275 | (0.272-0.278) | 0.103 | (0.100-0.107) |
|  | Pancreas | 0.129 | (0.121-0.137) | 0.077 | (0.063-0.091) |
|  | Liver | 0.262 | (0.249-0.276) | 0.127 | (0.101-0.152) |
|  | Lung | 0.228 | (0.223-0.233) | 0.096 | (0.088-0.103) |
|  | Esophagus | 0.223 | (0.204-0.242) | 0.110 | (0.080-0.141) |
|  | Stomach | 0.183 | (0.169-0.196) | 0.120 | (0.094-0.145) |
|  | Leukemia | 0.415 | (0.404-0.426) | 0.171 | (0.149-0.192) |
|  | Ovary | 0.260 | (0.244-0.276) | 0.078 | (0.054-0.102) |
|  | Bladder | 0.288 | (0.275-0.302) | 0.070 | (0.056-0.084) |
|  | Lymphoma | 0.356 | (0.346-0.365) | 0.127 | (0.112-0.142) |
|  | Uterus | 0.211 | (0.192-0.231) | 0.044 | (0.032-0.055) |
|  | Myeloma | 0.294 | (0.280-0.308) | 0.109 | (0.086-0.133) |
|  | Breast | 0.204 | (0.186-0.221) | 0.051 | (0.043-0.059) |
|  | Kidney | 0.173 | (0.161-0.184) | 0.042 | (0.030-0.055) |
|  | Rectum | 0.205 | (0.191-0.218) | 0.083 | (0.062-0.103) |
|  | Colon | 0.156 | (0.150-0.162) | 0.067 | (0.055-0.078) |
|  | Prostate | 0.127 | (0.116-0.138) | 0.041 | (0.035-0.047) |

a. Risk differences were calculated with non-EP as the reference group, through direct standardization based on the distribution of 5-year age group, sex, and stage by tumor sites in the total population.

b. 95% Confidence intervals were constructed using normal approximation.

Table S4a. Risk ratios (RRs) for one-year mortality comparing emergency presentation (EP) groups with non-EP by tumor site, with progressive covariate adjustment (Models 0–3). See Table S4b for Models 4–7.

|  |  | **Model 0 (unadjusted)** | **Model 1 (+ Sex, Age)** | **Model 2 (Model 1 + Stage)** | **Model 3 (Model 2 + Histology)** |
| --- | --- | --- | --- | --- | --- |
| Tumor Site | **EP Type** | **RR (95% CI)** | **RR (95% CI)** | **RR (95% CI)** | **RR (95% CI)** |
| Overall | EP-IP | 3.39 (3.37-3.41) | 3.06 (3.04-3.08) | 2.10 (2.09-2.11) | 1.80 (1.79-1.81) |
|  | EP-OP | 2.13 (2.10-2.15) | 2.01 (1.99-2.03) | 1.60 (1.58-1.61) | 1.42 (1.41-1.43) |
| Pancreas | EP-IP | 1.26 (1.25-1.28) | 1.23 (1.21-1.24) | 1.19 (1.18-1.20) | 1.17 (1.16-1.19) |
|  | EP-OP | 1.17 (1.14-1.19) | 1.16 (1.14-1.18) | 1.12 (1.10-1.14) | 1.10 (1.08-1.12) |
| Liver | EP-IP | 1.67 (1.64-1.71) | 1.62 (1.58-1.65) | 1.49 (1.46-1.52) | 1.49 (1.46-1.52) |
|  | EP-OP | 1.37 (1.32-1.43) | 1.36 (1.30-1.41) | 1.27 (1.22-1.32) | 1.27 (1.22-1.32) |
| Lung | EP-IP | 1.78 (1.77-1.80) | 1.75 (1.73-1.76) | 1.43 (1.42-1.44) | 1.38 (1.37-1.39) |
|  | EP-OP | 1.38 (1.36-1.40) | 1.37 (1.35-1.39) | 1.22 (1.20-1.23) | 1.19 (1.17-1.20) |
| Esophagus | EP-IP | 1.59 (1.54-1.64) | 1.53 (1.48-1.57) | 1.43 (1.39-1.47) | 1.42 (1.38-1.46) |
|  | EP-OP | 1.31 (1.24-1.38) | 1.29 (1.22-1.36) | 1.24 (1.18-1.30) | 1.23 (1.17-1.30) |
| Stomach | EP-IP | 1.69 (1.65-1.74) | 1.58 (1.54-1.63) | 1.43 (1.40-1.47) | 1.43 (1.40-1.47) |
|  | EP-OP | 1.47 (1.40-1.55) | 1.44 (1.37-1.52) | 1.32 (1.26-1.38) | 1.30 (1.25-1.36) |
| Leukemia | EP-IP | 3.01 (2.93-3.10) | 2.81 (2.73-2.89) | 2.81 (2.73-2.89) | 1.95 (1.89-2.00) |
|  | EP-OP | 1.85 (1.75-1.96) | 1.78 (1.69-1.89) | 1.78 (1.69-1.89) | 1.46 (1.38-1.53) |
| Ovary | EP-IP | 2.26 (2.17-2.35) | 1.96 (1.89-2.04) | 1.85 (1.78-1.93) | 1.71 (1.65-1.78) |
|  | EP-OP | 1.41 (1.31-1.52) | 1.32 (1.24-1.42) | 1.29 (1.20-1.37) | 1.23 (1.16-1.31) |
| Bladder | EP-IP | 2.89 (2.80-2.98) | 2.51 (2.43-2.59) | 2.07 (2.00-2.13) | 2.02 (1.95-2.08) |
|  | EP-OP | 1.46 (1.39-1.54) | 1.41 (1.34-1.49) | 1.34 (1.28-1.41) | 1.33 (1.26-1.40) |
| Lymphoma | EP-IP | 3.26 (3.17-3.35) | 2.97 (2.89-3.06) | 2.97 (2.89-3.06) | 2.95 (2.87-3.03) |
|  | EP-OP | 1.82 (1.72-1.92) | 1.74 (1.66-1.84) | 1.74 (1.66-1.84) | 1.75 (1.66-1.84) |
| Uterus | EP-IP | 5.29 (5.03-5.57) | 4.47 (4.23-4.72) | 2.21 (2.10-2.34) | 2.05 (1.94-2.17) |
|  | EP-OP | 1.94 (1.78-2.11) | 1.71 (1.57-1.86) | 1.43 (1.32-1.54) | 1.40 (1.30-1.51) |
| Myeloma | EP-IP | 2.66 (2.55-2.78) | 2.50 (2.39-2.61) | 2.50 (2.39-2.61) | 2.50 (2.39-2.61) |
|  | EP-OP | 1.62 (1.49-1.77) | 1.57 (1.45-1.70) | 1.57 (1.45-1.70) | 1.57 (1.45-1.70) |
| Breast | EP-IP | 9.42 (9.10-9.76) | 7.19 (6.91-7.47) | 2.52 (2.41-2.63) | 2.29 (2.19-2.39) |
|  | EP-OP | 3.34 (3.12-3.58) | 2.82 (2.63-3.02) | 1.72 (1.61-1.83) | 1.66 (1.55-1.77) |
| Kidney | EP-IP | 3.30 (3.18-3.44) | 2.83 (2.72-2.95) | 1.83 (1.76-1.90) | 1.83 (1.76-1.90) |
|  | EP-OP | 1.74 (1.63-1.87) | 1.66 (1.56-1.78) | 1.30 (1.23-1.38) | 1.29 (1.22-1.37) |
| Rectum | EP-IP | 2.86 (2.75-2.99) | 2.49 (2.38-2.60) | 2.00 (1.92-2.08) | 1.98 (1.90-2.06) |
|  | EP-OP | 1.85 (1.70-2.02) | 1.72 (1.58-1.86) | 1.48 (1.37-1.60) | 1.46 (1.35-1.57) |
| Colon | EP-IP | 2.36 (2.30-2.41) | 2.08 (2.04-2.13) | 1.73 (1.69-1.77) | 1.72 (1.69-1.76) |
|  | EP-OP | 1.75 (1.67-1.83) | 1.63 (1.56-1.71) | 1.37 (1.32-1.42) | 1.34 (1.29-1.40) |
| Prostate | EP-IP | 11.71 (11.28-12.16) | 5.62 (5.36-5.90) | 2.98 (2.84-3.13) | 2.72 (2.60-2.86) |
|  | EP-OP | 4.02 (3.77-4.29) | 2.75 (2.58-2.93) | 1.99 (1.87-2.12) | 1.94 (1.82-2.06) |

a. Healthcare status indicators include Charlson comorbidity index (categorized as 0, 1, 2, 3+) and Kim Frailty index (in deciles by tumor sites), based on claims in the 365 days prior to diagnosis.

Table S4b. Risk ratios (RRs) for one-year mortality comparing emergency presentation (EP) groups with non-EP by tumor site, with progressive covariate adjustment (Models 4–7). Continued from Table S4a.

| **Tumor Site** | **EP Type** | **Model 4 (Model 3 + Health Status)^a^** | **Model 5 (Model 4 + Healthcare Utilization)^b^** | **Model 6 (Model 5 + Individual Characteristics)^c^** | **Model 7 (Model 5 + Contextual Characteristics)^d^** |
| --- | --- | --- | --- | --- | --- |
|  |  | **RR (95% CI)** | **RR (95% CI)** | **RR (95% CI)** | **RR (95% CI)** |
| **Overall** | EP-IP | 1.70 (1.69-1.71) | 1.64 (1.63-1.65) | 1.63 (1.62-1.64) | 1.65 (1.64-1.66) |
|  | EP-OP | 1.40 (1.38-1.41) | 1.37 (1.35-1.38) | 1.36 (1.34-1.37) | 1.35 (1.34-1.36) |
| **Pancreas** | EP-IP | 1.15 (1.14-1.16) | 1.13 (1.12-1.14) | 1.13 (1.12-1.14) | 1.14 (1.12-1.15) |
|  | EP-OP | 1.09 (1.07-1.11) | 1.08 (1.06-1.10) | 1.08 (1.06-1.10) | 1.07 (1.05-1.10) |
| **Liver** | EP-IP | 1.43 (1.40-1.47) | 1.41 (1.38-1.45) | 1.42 (1.39-1.45) | 1.42 (1.39-1.45) |
|  | EP-OP | 1.23 (1.18-1.28) | 1.23 (1.18-1.28) | 1.22 (1.18-1.27) | 1.22 (1.17-1.27) |
| **Lung** | EP-IP | 1.34 (1.33-1.35) | 1.31 (1.30-1.32) | 1.31 (1.30-1.32) | 1.32 (1.31-1.33) |
|  | EP-OP | 1.17 (1.15-1.18) | 1.16 (1.14-1.17) | 1.15 (1.14-1.17) | 1.15 (1.13-1.16) |
| **Esophagus** | EP-IP | 1.36 (1.32-1.40) | 1.30 (1.26-1.34) | 1.29 (1.25-1.34) | 1.30 (1.26-1.34) |
|  | EP-OP | 1.21 (1.15-1.27) | 1.18 (1.12-1.24) | 1.17 (1.11-1.24) | 1.17 (1.11-1.24) |
| **Stomach** | EP-IP | 1.38 (1.35-1.42) | 1.32 (1.29-1.36) | 1.33 (1.29-1.36) | 1.33 (1.30-1.37) |
|  | EP-OP | 1.28 (1.22-1.34) | 1.24 (1.19-1.30) | 1.23 (1.18-1.29) | 1.22 (1.17-1.28) |
| **Leukemia** | EP-IP | 1.84 (1.78-1.89) | 1.80 (1.75-1.85) | 1.79 (1.74-1.84) | 1.81 (1.76-1.87) |
|  | EP-OP | 1.40 (1.33-1.48) | 1.39 (1.32-1.47) | 1.38 (1.31-1.46) | 1.38 (1.31-1.45) |
| **Ovary** | EP-IP | 1.56 (1.50-1.62) | 1.47 (1.42-1.53) | 1.47 (1.41-1.53) | 1.50 (1.45-1.57) |
|  | EP-OP | 1.19 (1.12-1.27) | 1.16 (1.09-1.24) | 1.16 (1.09-1.24) | 1.15 (1.08-1.23) |
| **Bladder** | EP-IP | 1.79 (1.73-1.85) | 1.66 (1.61-1.72) | 1.66 (1.60-1.72) | 1.69 (1.63-1.75) |
|  | EP-OP | 1.27 (1.21-1.33) | 1.23 (1.17-1.29) | 1.22 (1.16-1.28) | 1.21 (1.15-1.27) |
| **Lymphoma** | EP-IP | 2.55 (2.48-2.63) | 2.42 (2.35-2.50) | 2.40 (2.32-2.47) | 2.46 (2.39-2.54) |
|  | EP-OP | 1.62 (1.54-1.71) | 1.59 (1.51-1.67) | 1.57 (1.49-1.65) | 1.56 (1.48-1.64) |
| **Uterus** | EP-IP | 1.78 (1.68-1.88) | 1.58 (1.49-1.68) | 1.57 (1.48-1.66) | 1.60 (1.51-1.69) |
|  | EP-OP | 1.33 (1.24-1.43) | 1.24 (1.15-1.34) | 1.23 (1.15-1.33) | 1.24 (1.15-1.34) |
| **Myeloma** | EP-IP | 2.23 (2.13-2.33) | 2.10 (2.01-2.20) | 2.09 (2.00-2.19) | 2.13 (2.03-2.23) |
|  | EP-OP | 1.49 (1.37-1.61) | 1.45 (1.34-1.58) | 1.43 (1.32-1.56) | 1.43 (1.32-1.55) |
| **Breast** | EP-IP | 1.95 (1.86-2.04) | 1.69 (1.61-1.77) | 1.69 (1.62-1.77) | 1.76 (1.68-1.84) |
|  | EP-OP | 1.51 (1.41-1.61) | 1.38 (1.29-1.48) | 1.37 (1.28-1.46) | 1.35 (1.27-1.44) |
| **Kidney** | EP-IP | 1.67 (1.60-1.73) | 1.57 (1.51-1.63) | 1.57 (1.51-1.64) | 1.60 (1.53-1.66) |
|  | EP-OP | 1.25 (1.18-1.33) | 1.21 (1.15-1.29) | 1.22 (1.15-1.29) | 1.20 (1.13-1.27) |
| **Rectum** | EP-IP | 1.75 (1.67-1.83) | 1.64 (1.57-1.72) | 1.64 (1.56-1.71) | 1.70 (1.62-1.77) |
|  | EP-OP | 1.38 (1.28-1.50) | 1.35 (1.25-1.46) | 1.34 (1.24-1.45) | 1.32 (1.22-1.43) |
| **Colon** | EP-IP | 1.58 (1.55-1.61) | 1.49 (1.46-1.53) | 1.49 (1.46-1.52) | 1.51 (1.48-1.54) |
|  | EP-OP | 1.28 (1.23-1.33) | 1.25 (1.20-1.30) | 1.24 (1.19-1.29) | 1.22 (1.18-1.27) |
| **Prostate** | EP-IP | 2.16 (2.05-2.27) | 1.92 (1.82-2.02) | 1.90 (1.80-2.00) | 1.95 (1.85-2.05) |
|  | EP-OP | 1.70 (1.60-1.81) | 1.61 (1.52-1.72) | 1.60 (1.51-1.71) | 1.58 (1.49-1.69) |

a. Healthcare utilization variables include indicator for screening status, count of ED visits (categorized as 0, 1, 2, 3+), number of inpatient days (with quadratic and cubic terms), and number of E&M visits (in deciles by tumor sites) in the past year, based on claims in the 365 days prior to diagnosis.
b. Individual-level characteristics include partnership status, Medicaid eligibility, and race/ethnicity of patients.
c. Contextual characteristics include SEER region, Yost composite score for neighborhood SES based on tract-level attributes (in Quintiles by counties), county-level metropolitan status of residency, and year of diagnoses. Model 7 is the final model for interpretation based on the 5% Change-in-Estimate criterion.

Table S5. Adjusted risk ratios (RRs) comparing inpatient EP (EP-IP) and outpatient EP (EP-OP) with non-EP by tumor site and follow-up period. Estimates from modified Poisson regression models with robust standard errors, adjusted for age, sex, stage, histology, comorbidity, frailty, healthcare utilization, and contextual characteristics. Follow-up periods include 30-day, 90-day (conditional on 30-day survival), 365-day (conditional on 90-day survival), and 365-day unconditional.

| **Tumor Site** | **EP Type** | **30-day** | **90-day^a^** | **365-day^b^** | **365-day (unconditional)** |
| --- | --- | --- | --- | --- | --- |
|  |  | **RRs (95% CI)^c^** | **RRs (95% CI)^c^** | **RRs (95% CI)^c^** | **RRs (95% CI)^c^** |
| **Overall** | EP-IP | 3.88 (3.81-3.96) | 2.29 (2.25-2.32) | 1.51 (1.49-1.52) | 1.65 (1.64-1.66) |
|  | EP-OP | 1.95 (1.89-2.01) | 1.58 (1.54-1.62) | 1.28 (1.26-1.30) | 1.35 (1.34-1.36) |
| **Pancreas** | EP-IP | 2.12 (2.03-2.22) | 1.48 (1.42-1.53) | 1.12 (1.10-1.15) | 1.14 (1.12-1.15) |
|  | EP-OP | 1.41 (1.31-1.52) | 1.24 (1.17-1.32) | 1.07 (1.03-1.11) | 1.07 (1.05-1.10) |
| **Liver** | EP-IP | 3.75 (3.46-4.07) | 2.13 (1.99-2.28) | 1.30 (1.24-1.36) | 1.42 (1.39-1.45) |
|  | EP-OP | 1.83 (1.60-2.09) | 1.59 (1.43-1.77) | 1.16 (1.08-1.24) | 1.22 (1.17-1.27) |
| **Lung** | EP-IP | 2.99 (2.91-3.08) | 1.89 (1.85-1.93) | 1.26 (1.24-1.28) | 1.32 (1.31-1.33) |
|  | EP-OP | 1.62 (1.55-1.70) | 1.36 (1.31-1.41) | 1.12 (1.09-1.14) | 1.15 (1.13-1.16) |
| **Esophagus** | EP-IP | 3.67 (3.21-4.18) | 1.78 (1.62-1.96) | 1.23 (1.17-1.30) | 1.30 (1.26-1.34) |
|  | EP-OP | 1.90 (1.56-2.33) | 1.27 (1.09-1.47) | 1.18 (1.09-1.27) | 1.17 (1.11-1.24) |
| **Stomach** | EP-IP | 2.99 (2.71-3.31) | 1.64 (1.52-1.77) | 1.29 (1.23-1.35) | 1.33 (1.30-1.37) |
|  | EP-OP | 1.62 (1.37-1.93) | 1.47 (1.30-1.66) | 1.20 (1.11-1.30) | 1.22 (1.17-1.28) |
| **Leukemia** | EP-IP | 3.69 (3.44-3.97) | 2.36 (2.20-2.54) | 1.72 (1.64-1.81) | 1.81 (1.76-1.87) |
|  | EP-OP | 1.98 (1.75-2.23) | 1.59 (1.39-1.81) | 1.28 (1.17-1.41) | 1.38 (1.31-1.45) |
| **Ovary** | EP-IP | 2.59 (2.33-2.87) | 1.88 (1.70-2.07) | 1.40 (1.30-1.51) | 1.50 (1.45-1.57) |
|  | EP-OP | 1.32 (1.12-1.56) | 1.32 (1.13-1.53) | 1.11 (0.99-1.24) | 1.15 (1.08-1.23) |
| **Bladder** | EP-IP | 5.00 (4.37-5.72) | 2.80 (2.55-3.08) | 1.57 (1.49-1.65) | 1.69 (1.63-1.75) |
|  | EP-OP | 1.63 (1.33-2.00) | 1.38 (1.21-1.58) | 1.16 (1.09-1.24) | 1.21 (1.15-1.27) |
| **Lymphoma** | EP-IP | 6.26 (5.75-6.81) | 3.55 (3.31-3.80) | 2.07 (1.97-2.18) | 2.46 (2.39-2.54) |
|  | EP-OP | 2.54 (2.21-2.90) | 1.85 (1.64-2.07) | 1.39 (1.28-1.51) | 1.56 (1.48-1.64) |
| **Uterus** | EP-IP | 4.95 (4.03-6.07) | 2.44 (2.13-2.81) | 1.39 (1.27-1.51) | 1.60 (1.51-1.69) |
|  | EP-OP | 1.93 (1.47-2.53) | 1.27 (1.05-1.53) | 1.18 (1.07-1.30) | 1.24 (1.15-1.34) |
| **Myeloma** | EP-IP | 5.11 (4.43-5.90) | 2.95 (2.66-3.28) | 1.85 (1.73-1.99) | 2.13 (2.03-2.23) |
|  | EP-OP | 2.63 (2.09-3.30) | 1.52 (1.27-1.84) | 1.28 (1.14-1.44) | 1.43 (1.32-1.55) |
| **Breast** | EP-IP | 6.11 (5.28-7.06) | 2.70 (2.43-3.01) | 1.45 (1.35-1.55) | 1.76 (1.68-1.84) |
|  | EP-OP | 2.30 (1.88-2.81) | 1.93 (1.68-2.23) | 1.20 (1.10-1.32) | 1.35 (1.27-1.44) |
| **Kidney** | EP-IP | 3.42 (3.04-3.86) | 2.13 (1.94-2.35) | 1.47 (1.38-1.56) | 1.60 (1.53-1.66) |
|  | EP-OP | 1.57 (1.31-1.88) | 1.47 (1.29-1.67) | 1.09 (0.99-1.19) | 1.20 (1.13-1.27) |
| **Rectum** | EP-IP | 4.36 (3.76-5.05) | 2.45 (2.19-2.74) | 1.44 (1.34-1.54) | 1.70 (1.62-1.77) |
|  | EP-OP | 1.88 (1.48-2.39) | 1.75 (1.46-2.09) | 1.21 (1.08-1.36) | 1.32 (1.22-1.43) |
| **Colon** | EP-IP | 2.83 (2.67-3.00) | 1.79 (1.70-1.89) | 1.34 (1.29-1.39) | 1.51 (1.48-1.54) |
|  | EP-OP | 1.61 (1.45-1.78) | 1.42 (1.29-1.55) | 1.14 (1.07-1.22) | 1.22 (1.18-1.27) |
| **Prostate** | EP-IP | 5.17 (4.42-6.05) | 2.66 (2.34-3.02) | 1.74 (1.63-1.86) | 1.95 (1.85-2.05) |
|  | EP-OP | 2.41 (1.98-2.95) | 1.77 (1.51-2.08) | 1.50 (1.38-1.62) | 1.58 (1.49-1.69) |

a. The 90-day risk period is conditional survival through the first 30 days.
b. This 365-day risk period is conditional survival through the first 90 days.
c. Confidence intervals were estimated based on robust standard errors.
